# The non-metabolic function of 6PGD coordinates CCNA2 and HMGA2 expression to drive colorectal cancer progression and drug response

**DOI:** 10.1186/s13046-025-03450-3

**Published:** 2025-07-03

**Authors:** Mingming Sun, Qi Yan, Xinru Zhai, Xintong Dai, Chenxin Yang, Huifang Zhao, Sizhen Lai, Jiyan Wang, Lian Li, Zhen Li, Yanping Li, Taoyuan Wang, Tao He, Jun Xue, Zhenghu Jia, Chunze Zhang, Shuai Zhang, Changliang Shan

**Affiliations:** 1 State Key Laboratory of Medicinal Chemical Biology, College of Pharmacy and Tianjin Key Laboratory of Molecular Drug Research, Nankai University, Tianjin, 300350 China; 2 School of Integrative Medicine, Tianjin University of Traditional Chinese Medicine, Tianjin, 301617 China; 3 State Key Laboratory of Medicinal Chemical Biology, College of Life Sciences, Nankai University, Tianjin, 300071 China; 4 KingMed School of Laboratory Medicine, Guangzhou Medical University, Guangzhou, 510180 China; 5 Department of Pathology, Institute of Precision Medicine, Jining Medical University, Jining, 272067 China; 6 Cardiothoracic Surgery Department, Characteristic Medical Center of the Chinese People’s Armed Police Force, Tianjin, 300162 China; 7 Department of Pathology, Characteristic Medical Center of The Chinese People’s Armed Police Force, Tianjin, 300162 China; 8 Department of General Surgery, The First Affiliated Hospital of Hebei North University, Zhangjiakou, 075000 China; 9 The First Affiliated Hospital, Biomedical Translational Research Institute, Guangdong Province Key Laboratory of Molecular Immunology and Antibody Engineering, Jinan University, Guangzhou, 510632 China; 10 Tianjin Key Laboratory for Modern Drug Delivery & High-Efficiency, Collaborative Innovation Center of Chemical Science and Engineering, School of Pharmaceutical Science and Technology, Tianjin University, Tianjin, 300193 China; 11 Department of Colorectal Surgery, Tianjin Union Medical Center, Nankai University, Tianjin, 300121 China; 12 Tianjin International Joint Academy of Biomedicine, Tianjin, 300457 China

**Keywords:** Colorectal cancer (CRC), 6-phosphogluconate dehydrogenase (6PGD), Non-metabolic activity, Proliferation, Metastasis

## Abstract

Emerging evidence demonstrates that the metabolic and non-metabolic functions of metabolic enzymes play a key role in tumorigenesis and progression. 6-phosphogluconate dehydrogenase (6PGD) is a key metabolic enzyme in the pentose phosphate pathway (PPP), which displays aberrant expressions and functions in cancer. However, whether 6PGD serves non-metabolic functions in regulating cancer progression and drug response has not been reported. Here, we found that highly expressed 6PGD contributes to colorectal cancer (CRC) tumor growth and tumor metastasis. Mechanistically, 6PGD binds to ALKBH5 and inhibits its activity through the non-metabolic activity of 6PGD; this increases m^6^A modification levels and the stability of MDM2 mRNA and decreases the p53 protein stability, subsequently activating the expression of CCNA2 and HMGA2, which are responses to CRC tumor growth and tumor metastasis. Collectively, these findings reveal the multi-functionality of 6PGD in promoting CRC tumor growth, tumor metastasis, and drug responses through its non-metabolic activity.

**Supplementary Information:**

The online version contains supplementary material available at 10.1186/s13046-025-03450-3.

## Introduction

Colorectal cancer (CRC) is a major cause of cancer deaths, ranking third in terms of global cancer-related morbidity and mortality [1]. At present, the treatment of CRC mainly includes surgery, chemoradiotherapy, and targeted drug therapy. The continuous development of 5-fluorouracil (5-FU) and oxaliplatin have precipitated immense progress in developing drug treatments for CRC. However, acquired resistance to 5-FU or oxaliplatin has resulted in cancer recurrence and metastasis, which are main causes of CRC-related deaths [2, 3]. In addition, the molecular mechanisms underlying CRC progression and chemotherapy responses have not been fully clarified. Thus, there is a critical need not only to elucidate molecular mechanisms underlying CRC carcinogenesis, but also to develop effective strategies to overcome 5-FU or oxaliplatin resistance and prevent tumor recurrence or metastasis.

An increasing volume of studies have reported that metabolic enzymes play an indispensable role in metabolic reprogramming, and abnormal expression or activity is closely related to cancer progression and chemotherapy sensitivity [4, 5]. Notably, recent studies have found that some metabolic enzymes serve non-classical/non-metabolic functions and are involved in the regulation of cell cycles, DNA damage repair, cell proliferation, apoptotic pathways, and tumor microenvironments, thus affecting the occurrence and progression of tumors and therapeutic efficacy [6–9]. For example, when pyruvate kinase M2 (PKM2) enters the nucleus, it binds with β-catenin as a transcriptional regulator to promote the expression of the c-Myc gene, thereby promoting the expression of genes related to glycolysis and enhancing glucose uptake and lactic acid production [10, 11]. Alpha-enolase (ENO1) serves as a non-canonical RNA-binding protein (RBPs) to regulate iron regulator protein 1 (IRP1) expression and promote yes-associated protein 1 (YAP1) translation [12]. Thus, interventions in these metabolic enzymes’ activity is not an effective mean to treat tumors and significantly improve the sensitivity of tumor cells to chemotherapy drugs.

6-phosphogluconate dehydrogenase (6PGD) is the third key enzyme of the pentose phosphate pathway (PPP), which is strongly expressed and highly active in various tumors [13]. Our previous studies demonstrated that YTH domain family 2 (YTHDF2) promotes lung cancer cell growth by facilitating 6PGD mRNA translation [14]; lysine acetylation and arginine methylation modification increase 6PGD activity [15, 16]. Thus, the 6PGD is a potential target for treating cancer. We have screened specific small molecule inhibitor Physcion, which significantly inhibits tumor growth and can also reverse cisplatin resistance in ovarian and lung cancer by targeting 6PGD [17, 18]. However, whether 6PGD serves a non-metabolic function in regulating cancer development and drug responses has not been reported. In this study, we discovered that 6PGD exhibits non-metabolic activity, including a previously unknown function as a regulator in CRC tumor growth and metastasis.

In the current study, we demonstrated that 6PGD is more strongly expressed in CRC and that it increases cyclin A2 (CCNA2) and high-mobility group AT-hook 2 (HMGA2) expression, thereby promoting the CRC cell cycles and cell migration. Furthermore, we uncovered the underlying mechanism of 6PGD in promoting tumor growth and metastasis, which binds to ALKBH5 and inhibits the activity of ALKBH5, resulting in the increase of m^6^A modifications of MDM2 mRNA and the subsequent increase of its mRNA stability mediated by YTHDF2. Then, MDM2 decreases the p53 protein stability, which leads to increased CCNA2 and HMGA2 expression in a 6PGD non-metabolic manner. Thus, targeting the non-metabolic activity of 6PGD enhances chemotherapy sensitivity in CRC. Collectively, our results demonstrate that the non-metabolic activity of 6PGD regulates ALKBH5 and promotes global m^6^A modification in CRC, adding another dimension to the regulation of CRC tumor growth and metastasis through the MDM2-CCNA2/HMGA2 axis in a non-metabolic manner.

## Materials and methods

### Cell culture

SW480, RKO, LoVo, HCT116, SW620, HT-29, and HCT8 cell lines were cultured in RPMI1640 medium (Thermo Fisher Scientific, USA) supplemented with 10% fetal bovine serum (FBS, ExCell Bio, China). HEK293T cell was maintained in Dulbecco’s modified Eagle’s medium (DMEM, Thermo Fisher Scientific, USA) supplemented with 10% FBS. Normal proliferating Human normal colonic epithelial cells (NCM460) was cultured in RPMI1640 medium with 10% FBS.

### Cell proliferation assay

The impact of 6PGD on cell proliferation were detected by cell counting assay, then the control and 6PGD knocked down (or 6PGD WT / 6PGD K76R overexpressed ) cells were seeded in 24-well plates. Cell growth was determined by cell numbers recorded at 0, 1, 2, 3 and 4 days after being seeded. As for the effect of Physcion, CRC cells were seeded in 24-well plates. On the second day of seeding the cells, the appropriate concentration of Physcion was added to the medium, and the cell count was started on the third day and continued for four days. For chemotherapy sensitivity assay, the control and 6PGD knocked down cells were seeded in 24-well plates. On the second day of seeding the cells, the appropriate concentration of chemotherapy drugs was added to the medium, and the cell number was recorded on the fifth day.

### Colony formation

The cells were plated in 6-well plates for 1500 cells and cultured for 2 weeks. The colonies were calculated after fixed and stained with 1% crystal violet. Colony number was measured by Image J.

### Western blot analysis

Cells were lysed with NP40 lysis buffer (150 mM NaCl, 10 mM HEPES [pH = 7.0], 1%NP40, 5 mM Na_4_P_2_O_7_, 5 mM NaF, 2 mM Na_3_VO_4_) containing protease inhibitor (complete ULTRA Tablets, Min, EDTA-free, EASYpack, Roche) on ice for 30 min and then centrifuged at 12,000 rpm for 15 min at 4 °C. Protein samples were separated by 8% or 10% SDS-PAGE and transferred onto 0.2–0.45 μm PVDF membranes (Millipore, USA) according to protein molecular mass. Firstly, the membranes were blocked with 5% non-fat milk for 2 h. Secondly, the membranes incubated overnight at 4 °C (or 2 h at room temperature) with the primary antibody. Thirdly, the membranes incubated 1 h at room temperature with secondary antibody. Lastly, the signals were detected using Luminol substrate solution (Millipore, USA).

### Clinical samples

26 paired frozen clinical colorectal cancer tumor tissues and adjacent non-tumor colorectal tissues (Cohort 1 = 26) and 30 paired colorectal cancer (CRC) tissue microarrays (Cohort 2 = 30 and Cohort 3 = 72) which included 30 (or 72) CRC tissues and 30 (or 72) adjacent non-tumor colorectal tissues were collected from Tianjin Union Medical Center (Tianjin, China) after surgical resection. The clinical CRC specimens all have the written consent approving the use of the samples for research purposes from patients. The relevant characteristics were shown in Supplementary Table. The study protocol was approved by the Institute Research Ethics Committee at Nankai University.

### Tumor formation in nude mice

Cells were harvested by trypsinization, washed twice with sterile PBS and resuspended at 50 × 10^6^ cells/mL. Then, 0.2 mL aliquots were injected subcutaneously into female nude mice with HCT116 cells harboring empty vector on the left flank, and cells with stable knockdown of endogenous 6PGD on the right flank. Tumor growth was recorded by measurement of two perpendicular diameters using the formula 4π/3 x (length/2) x (width/2)^2^. The tumors were harvested and weighed at the experimental endpoint, and the masses of tumors (g) derived from cells with and without stable knockdown of endogenous 6PGD. Tumor tissues were then subjected to gradient dehydration, sectioned, embedded in paraffin, and stained with IHC for histological examination. Statistical analyses have been done by comparison in relation to the control group with a two-tailed paired Student’s t test.

### Patient-derived organoid (PDO) models

The fresh CRC tumor tissues used to establish the PDO model were provided from Tianjin Union Medical Center (Tianjin, China). Fresh tissues were digested with digestive fluid and then filtered with cellular mesh. Matrix glue and organoid medium was added to the filtered tissue and cultured in a cell incubator until the tissue diameter reached to 30–50 μm. A certain number of organoids were inoculated on 96-well cell culture plates, and the fluid was changed daily. After 5 days, the lentivirus medium containing 6PGD was replaced daily. After 5 days of treatment, cell viability was measured by CellTiter-Glo3D method, organoids were collected, and IHC experiment was used to verify whether 6PGD was knocked down.

### Patient-derived xenograft (PDX) models

The fresh CRC tumor tissues used to establish the PDX mice model were provided from Tianjin Union Medical Center (Tianjin, China). The tumor sample were cut into approximately 1 mm^3^, and the tissues was pushed under skin of mice by TROCHAR. When tumor volumes reached approximately 100 mm^3^, mice were randomly distributed into groups of 7 mice. To investigate the effect of 6PGD on tumorigenicity of CRC, 6PGD shRNA or control shRNA virus was injected precisely into the center of the xenografted tumors first three days for three times. Tumor tissues were then subjected to gradient dehydration, sectioned, embedded in paraffin, and stained with IHC for histological examination. Statistical analyses have been done by comparison in relation to the control group with a two-tailed paired Student’s t test.

To explore the effect of 5-FU and Oxaliplatin on tumorigenicity of CRC, mice were received vehicle control, 5-FU (5 mg/kg), or Oxaliplatin (4 mg/kg), and they were administered every three days by intraperitoneal injection (100 µL). The mice were sacrificed, and the tumors were excised, imaged and weighed after drugs treatments. Tumor tissues were then subjected to gradient dehydration, sectioned, embedded in paraffin, and stained with IHC for histological examination. Statistical analyses have been done by comparison in relation to the control group with a two-tailed unpaired Student’s t test.

### Lung metastasis model in nude mice

For lung metastasis experiments, nude mice were randomly divided into two groups (*n* = 9 per group). Cells were harvested by trypsinization, washed twice with sterile PBS and resuspended at 50 × 10^6^ cells/mL. Then, 0.1 mL aliquots were injected via the tail vein with 5 × 10^6^ HCT116 cells harboring empty vector or cells with stable knockdown of endogenous 6PGD. After four weeks, nude mice were sacrificed by cervical dislocation. Lung tissue was removed, and imaged, and the number of nodules on the surface of the lung was recorded to assess tumor metastasis. Lung tissues were then subjected to gradient dehydration, sectioned, embedded in paraffin, and stained with H&E for histological examination.

### Primary colorectal cancer model

Colitis associated colon cancer model was induced as previously described by Endharti et al. ^1^. Colorectal cancer was induced by intraperitoneal injection of a single dose of the mutagenic agent AOM (10 mg/kg, Sigma Aldrich) on day 1 followed by 3 cycles of 2.5% DSS in drinking water for 1 week and normal drinking water for 2 weeks. Prior to 2.5% DSS treatment, the mice were injected with concentrated 6PGD shRNA or control shRNA virus for three consecutive days by I.V. Mice were sequentially killed randomly at the end of the 10th week. Colon tissues were collected for analysis by Western blot.

### Bioinformatics analysis

Kyoto Encyclopedia of Genes and Genomes (KEGG) pathway analysis and Gene Ontology (GO) analysis were performed by *DAVID Bioinformatics* (https://david.ncifcrf.gov/tools.jsp). The data of KEGG and GO were graphed using *Bioinformatics* (https://www.bioinformatics.com.cn/).

### Statistical analysis

GraphPad Prims 8 software was used to analyze data. Statistical details of experiments including statistical tests, mean ± SD. The differences between two groups with similar variances were analyzed using a two-tailed Student’s t test. All statistical analyses were performed using GraphPad Prism 8 software. A *p* value lower than 0.05 was considered significant.

## Results

### 6PGD expression is elevated in CRC

**Fig. 1:**
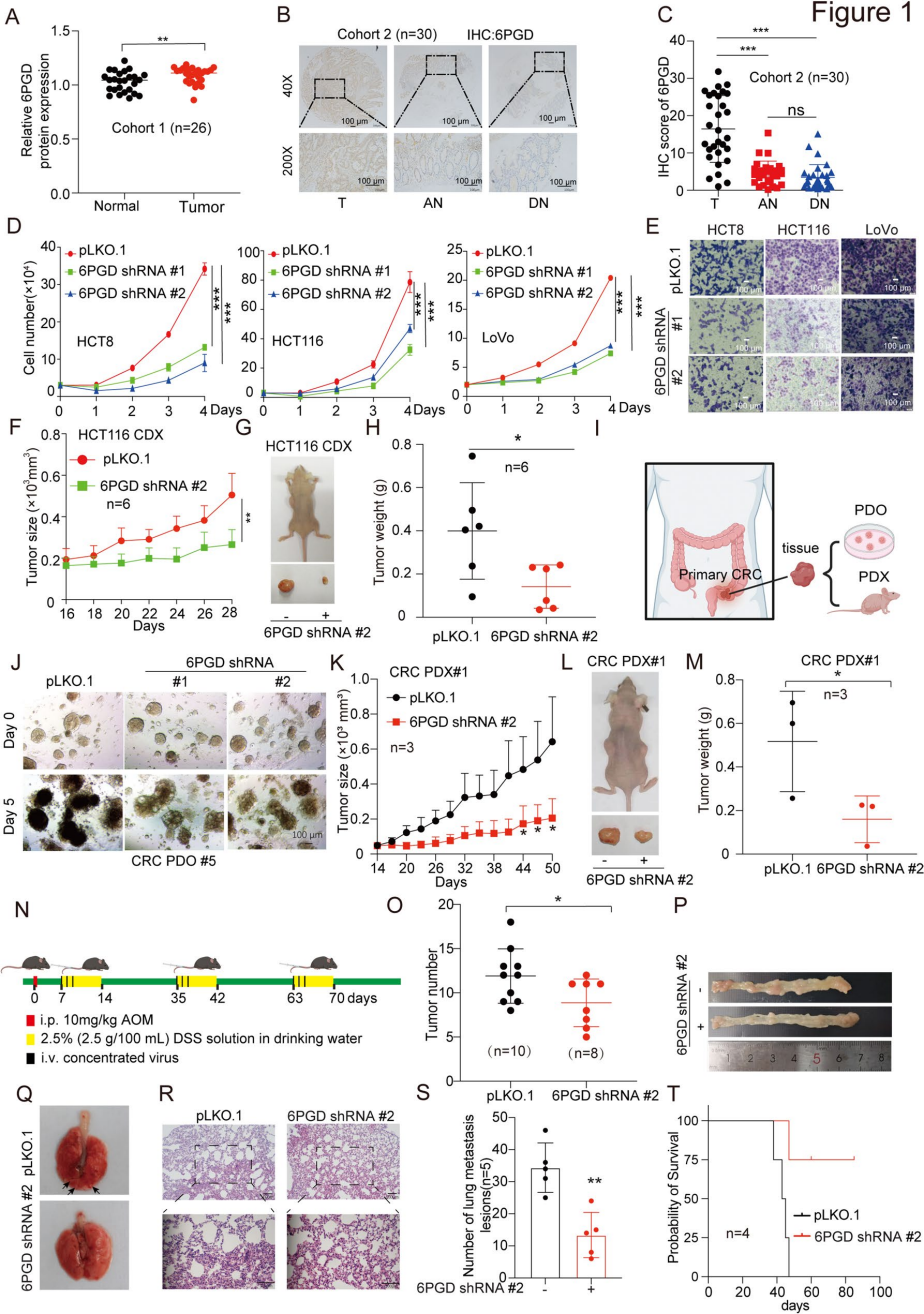
6PGD expression is elevated in CRC and required for colorectal cancer cell growth and metastasis. (**A**) The 6PGD protein levels in CRC tumor tissues (T) and matched adjacent normal tissues (N) were determined by western blotting. (Cohort 1: *n* = 26). (**B**) The expression of 6PGD was determined in CRC tissues microarray (TMA), which containing CRC tumor tissues (T), adjacent normal tissues (AN) and distant normal tissues (DN) (Cohort 2: *n* = 30) by immunohistochemical (IHC) stating assay. (**C**) Quantification of 6PGD expression by Image J software from IHC data. (**D**) Cell proliferation was determined in HCT8, HCT116 and LoVo cells with stable knockdown of 6PGD by cell number counting assay. (**E**) Cell migration was determined in HCT8, HCT116, and LoVo cells with stable knockdown of 6PGD by Transwell invasion assay. (**F**) Tumor growth was compared between xenograft nude mice injected with 6PGD knockdown and control vector of HCT116 cells (*n* = 6). (**G**) Dissected tumors in a representative nude mouse are shown. (**H**) Tumor mass in xenograft nude mice injected with knockdown of 6PGD HCT116 cells and control vector cells. (**I**) A schematic model shows how to build CRC PDO and PDX. (**J**) A representative PDO#5 are shown. (**K**) Tumor growth was compared between xenograft nude mice bearing with CRC PDX#1 injected with 6PGD shRNA virus and control shRNA virus (*n* = 3). (**L**) Dissected tumors in a representative nude mouse are shown (PDX#1). (**M**) Tumor mass in xenograft nude mice injected with 6PGD shRNA virus and control shRNA virus (PDX#1). (**N**) A schematic model shows how to induce CRC mouse model. (**O**) Tumor number in induced CRC mouse model. (**P**) Dissected colorectal tissue in induced CRC mouse model. (**Q**-**R**) Dissected lung and H&E staining of metastatic foci in lung paraffin sections from tail vein-injected BALB/c nude mice with knockdown of 6PGD HCT116 cells and control vector cells for 30 days (*n* = 5). (**S**) The quantification of metastatic foci in mouse lung. (**T**) Survival curve of tail vein-injected BALB/c nude mice with knockdown of 6PGD and control vector HCT116 cells (*n* = 4). The data represent mean values ± SD from three replicates of each sample (*0.01 < *p* < 0.05; **0.001 < *p* < 0.01; ***p < 0.001)

To explore the role of 6PGD in CRC-related tumorigenicity and chemotherapy response, we analyzed the expression of 6PGD in CRC samples. Firstly, we found that the expression levels of 6PGD were higher in CRC tissues based on the GEPIA data (Fig. S1A). Furthermore, the elevated expression of 6PGD predicted poor prognosis in CRC (Fig. S1B). To validate the clinical relevance of our findings based on a public database, we analyzed the 6PGD expression in a sample of 26 CRC patients (cohort 1: *n* = 26, CRC tissues (T), paired adjacent normal tissues (N)). Quantitative real-time PCR (qRT-PCR) and western blotting consistently showed that 6PGD mRNA and protein levels were significantly higher in CRC tumor tissues than in adjacent non-tumor colorectal tissues (Fig. S1C, Fig. 1A, and Fig. S1D). In addition, we also performed immunohistochemistry (IHC) staining to determine the expression of 6PGD protein in CRC tumor tissue microarrays (cohort 2: *n* = 30, CRC tissues (T), adjacent normal colon (AN), distant normal colon (DN)). IHC staining showed that the expression of 6PGD was positive in 26 (86.7%) and strongly positive in 14 (46.7%) of the 30 patients with cancer (Supplementary Table 1), and it was significantly higher in CRC tumor tissues than in paired adjacent normal tissues (Fig. 1B and C). However, no correlation was observed between tumor grade and 6PGD levels (Supplementary Table 2). In addition, the higher expression of 6PGD in CRC tumor tissues than in paired adjacent normal tissues were validated in another CRC tumor tissue microarrays (cohort 3: *n* = 72) (Fig. S1E and S1F). Lastly, we also assessed the expression of 6PGD in various human CRC cells, including SW480, RKO, LoVo, HCT116, SW620, HT-29, and HCT8 compared to normal proliferating colonic epithelial cells (NCM460); we then found that 6PGD was highly expressed in most CRC cells (Fig. S1G). Together, these data demonstrated that 6PGD was highly expressed in CRC and correlated with overall survival, suggesting that 6PGD is a promising anti-cancer target.

### 6PGD promotes CRC cell proliferation and migration in vitro

To elucidate the role of 6PGD in CRC, we generated stable 6PGD knockdown or knock out cells using specific short hairpin RNAs (shRNAs) or single guide RNA (sgRNA). We found that the knockdown or knock out of 6PGD decreased HCT8, HCT116 and LoVo cells’ proliferation and colony formation (Fig. 1D and Fig. S1H-S1I). While, exogneous expression of 6PGD promoted HCT116 and HCT8 cells’ proliferation (Fig. S1J). In addition, we also used siRNA to transient knockdown 6PGD and found that 6PGD knockdown decreased cell proliferation (Fig. S1K-S1L). What’s more, the cell proliferation and colony formation were also decreased in HCT8, HCT116, and LoVo cells treated with 6PGD inhibitor Physcion (Fig. S1M-S1N). These findings showed that 6PGD promotes CRC cell proliferation in vitro. We also explored the effects of 6PGD on cell migration, and found that targeting 6PGD inhibited CRC cell migration (Fig. 1E, Fig. S1N-S1V). Taken together, these results suggested that the expression and activity of 6PGD promote CRC cell proliferation and migration in vitro.

### 6PGD promotes CRC tumor growth and lung metastasis in vivo

The impact of 6PGD on CRC tumor growth in vivo was also explored. In a xenograft experiment in which nude mice were injected with control and 6PGD knockdown HCT116 cells on the left and right flanks, respectively, we found that the knockdown of 6PGD inhibited the tumor growth rate, decreased tumor size (Fig. 1F and 1G), and diminished tumor masses (Fig. 1H) compared with the control group. Furthermore, we constructed CRC patient-derived organoid (PDO) and patient-derived xenograft (PDX) models using fresh clinical CRC tumor tissues to explore the role of 6PGD in CRC (Fig. 1I). In the two PDO models, the effect of 6PGD on CRC tumor growth was examined after 5 days of continuous infection with 6PGD shRNA lentivirus. We found that the knockdown of 6PGD inhibited PDO tumor growth (Fig. S2A-S2B and Fig. 1J). The PDO morphology was determined using a H&E staining assay to confirm that PDO have been successfully constructed; we also applied the IHC staining assay to confirm the 6PGD knockdown efficiency and found that the knockdown of 6PGD decreased the cell proliferation marker Ki-67 in PDO (Fig. S2C).

Meanwhile, we applied two PDX models to validate the effect of 6PGD on tumor growth through an intratumoral 6PGD shRNA lentivirus injection. We found that the knockdown of 6PGD decreased the tumor growth rate (Fig. 1K and Fig. S2D, S2H). Moreover, the xenograft tumor size was smaller than that for the control group (Fig. 1L and Fig. S2E, S2F, S2I), and the tumor weight was also reduced (Fig. 1M and Fig. S2G, S2J). The 6PGD knockdown efficiency was confirmed by an IHC staining assay; additionally, the knockdown of 6PGD decreased the cell proliferation marker Ki-67 level in tumors (Fig. S3J). To further clarify the role of 6PGD in the development of CRC, we applied an azoxymethane/dextran sulfate sodium-(AOM/DSS-) induced CRC model in mice and simultaneously injected a 6PGD shRNA or control lentivirus through the tail vein to knockdown 6PGD in mice (Fig. 1N). Compared with the control group, the number of CRC tumors in the knockdown of 6PGD group was significantly reduced (Fig. 1O and P), while there was no effect on the length of the small intestine and colorectal (Fig. S2K-S2N). The 6PGD knockdown efficiency was confirmed via a western blotting assay; we also found that the 6PGD protein levels were significantly higher in colorectal tumor tissues than in adjacent non-tumor colorectal tissues (Fig. S2O). Collectively, these data indicated that 6PGD promotes CRC tumor growth in vivo.

Lastly, the impact of 6PGD on CRC lung metastasis and survival of mice in vivo were also explored. Firstly, the knockdown of 6PGD and control HCT116 cells were transplanted into mice via tail-vein injection (*n* = 9). Firstly, we taken five mice to examine the effects of 6PGD on lung metastasis, and we found that the knockdown of 6PGD decreased the formation of metastasis nodes on lungs over a 30-day period (*n* = 5) (Fig. 1Q and 1S). Then, the four mice left to examine the effects of 6PGD on the survival of mice and found that the knockdown of 6PGD can prolong the survival of mice (*n* = 4) (Fig. 1T). Collectively, these data indicated that 6PGD promotes CRC lung metastasis in vivo.

### The non-metabolic activities of 6PGD promotes CRC tumor growth and lung metastasis

**Fig. 2:**
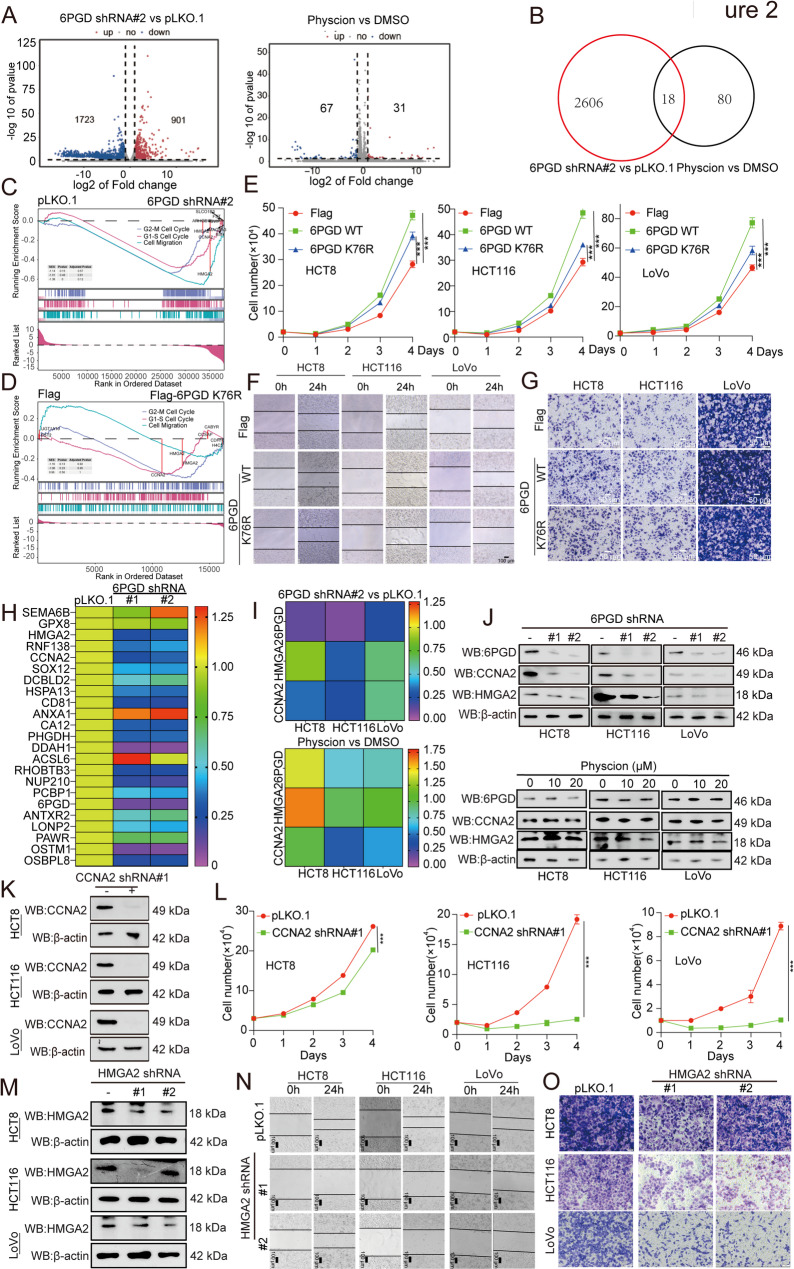
6PGD promotes CRC progression in a catalytic-activity-independent manner. (**A**) *left*: Volcano plot showing the differentially depleted (blue) and enriched (red) genes in HCT116 cells with stable knockdown 6PGD. *Right*: Volcano plot showing the differentially depleted (blue) and enriched (red) genes in HCT116 cells treated with or without Physcion. (**B**) Venn diagram showing the shared down-regulation between 6PGD knockdown-related down-regulation genes and Physcion treatment-related down-regulation genes. A total of 18 shared down-regulationgenes were observed. (**C**) The cell cycles and cell migration pathways riched in 6PGD related pathway were analyzed by gene set enrichment analysis (GSEA) based on knockdown of 6PGD and control cells RNA-seq. (**D**) The cell cycles and cell migration pathways riched in 6PGD related pathway were analyzed by gene set enrichment analysis (GSEA) based on over-expression of 6PGD K76R and control cells RNA-seq. (**E**-**G**) The cell proliferation (**E**), cell migration (**F**), and cell invasion (**G**) were examined in the CRC cells with exogenous expression of 6PGD WT or 6PGD K76R. (**H**) Fold change in mRNA levels of the top 23 genes based on RNA-seq were determined by qRT-PCR assays. (**I**-**J**) The expression of 6PGD, CCNA2, and HMGA2 were examined in the knockdown of 6PGD cells or Physcion treated cells by qRT-PCR and western blotting. (**K**) The expression of CCNA2 was determined in knockdown of CCNA2 HCT8, HCT116, and LoVo cells by western blotting. (**L**) Cell proliferation was determined in the HCT8, HCT116, and LoVo cells with stable knockdown of CCNA2 by cell number counting assay. (**M**) The expression of HMGA2 was determined in the HCT8, HCT116, and LoVo cells with stable knockdown of HMGA2 by western blotting. (**N**) Cell migration was determined in the knockdown of HMGA2 HCT8, HCT116, and LoVo cells by Wound-healing assay. (**O**) Cell invasion was determined in the knockdown of HMGA2 HCT8, HCT116, and LoVo cells by Transwell invasion assay. The data represent mean values ± SD from three replicates of each sample (*0.01 < *p* < 0.05; **0.001 < *p* < 0.01; ***p < 0.001)

To explore the molecular mechanism behind 6PGD’s regulation of CRC tumor growth and lung metastasis, we performed RNA sequencing (RNA-seq) and found 1,723 genes to be down-regulated and 901 genes to be up-regulated in the 6PGD knockdown group. However, only 67 genes were down-regulated, and 31 genes were up-regulated in the Physcion-treated group (Fig. 2A). In all, only 18 genes were jointly regulated by the knockdown of 6PGD and treated by Physcion (Fig. 2B). These results mean that many genes were independent of the metabolic activity of 6PGD. Thus, we focus on the mechanisms behind the non-metabolic activities of 6PGD in regulating CRC tumor growth and cell migration. We performed the gene set enrichment analysis (GSEA) based on the knockdown of 6PGD and Physcion-treated cells’ RNA sequencing. Unexpectedly, the GSEA results showed that the knockdown of 6PGD significantly altered cell cycles and cell migration signaling pathways (Fig. 2C); conversely, targeting 6PGD with Physcion had no effect on cell cycles and cell migration signaling pathways (Fig. S3A-S3B). Furthermore, we performed 6PGD wildtype (6PGD WT) and catalytically inactive 6PGD (6PGD K76R) RNA-seq to identify pathways triggered independent of its metabolic function. Then we performed GSEA to analyze the pathway were co-rich in overexpression of 6PGD WT or K76R 6PGD, knockdown of 6PGD, and Physcion-treated cells, and found that the co-regulated gene were riched in cell cycles and cell migration signaling pathways excepted for the Physcion-treated cells (Fig. S3B). Lastly, we performed the GSEA based on the 6PGD WT or K76R 6PGD cells’ RNA sequencing. Unexpectedly, the GSEA results also showed that the 6PGD WT or K76R 6PGD significantly altered cell cycles and cell migration signaling pathways (Fig. 2D and Fig. S3C).

To validate the RNA sequencing results, we examined the effect of 6PGD on CRC cell cycles and found that the knockdown of 6PGD significantly inhibited the CRC cell cycles (Supplementary Table 3). We explored the non-metabolic activity of 6PGD in relation to whether it drives cell proliferation, the cell cycles, and cell migration and invasion in CRC. Thus, we examined the effect of catalytically inactive 6PGD (6PGD K76R) on CRC cell proliferation, the cell cycles, and cell migration and invasion. We found that 6PGD K76R exerts a similar effect as 6PGD WT on cell proliferation (Fig. 2E), the cell cycles (Supplementary Table 4), and cell migration and invasion (Fig. 2F and 2G, and Fig. S3D). In addition, the reduction of cell proliferation (Fig. S3E), the cell cycles (Supplementary Table 5), cell migration (Fig. S3F), and cell invasion (Fig. S3G and Fig. S3H) were also partly rescued via the exogneous expression of 6PGD WT or 6PGD K76R in the knockdown of 6PGD cells. These data together suggested that the non-metabolic activities of 6PGD regulate CRC tumor growth mediated by the regulation of cell cycles and cell migration.

### 6PGD increases the CCNA2 and HMGA2 expression independent of metabolic activity to promote tumor growth and lung metastasis

Subsequently, we investigated how the non-metabolic activities of 6PGD regulate cell cycles and cell migration. We analyzed and confirmed the top 23 genes that are the most down-regulated in the knockdown of 6PGD cells via qRT-PCR assay (Fig. 2H) and found that CCNA2 and HMGA2 were related to cell cycles and cell migration; this was associated with decreased mRNA and protein levels in the knockdown or knock out of 6PGD cells but not in 6PGD inhibitor Physcion-treated cells (Fig. 2I and 2J, and Fig. S3I). We also detected the expression of CCNA2 and HMGA2 by IHC stating assay in CDX and PDX model tumor tissues and found that the knockdown of 6PGD decreased the expression of CCNA2 and HMGA2 proteins (Fig. S3J). Collectively, these data suggested that 6PGD promotes CRC cell cycles and cell migration in a non-metabolic-activity-dependent manner mediated by regulating CCNA2 and HMGA2 expression.

### CCNA2 and HMGA2 expression promotes cell proliferation and cell migration

CCNA2 is an established regulator of cell proliferation and has been used for molecular diagnostics as a proliferation marker. Here, we would like to explore the impact of its expression in CRC. IHC staining showed that it was significantly higher in CRC than paired adjacent normal tissues (Cohort 2: *n* = 30 and Cohort 3: *n* = 72) (Fig. S3K-S3N, Supplementary Tables 6-Table 7). In addition, we analyzed CCNA2 expression based on the GEPIA database and found that CCNA2 is highly expressed in CRC tissues compared to adjacent tissues (Fig. S3O). Moreover, the expression of CCNA2 protein was positively correlated with 6PGD expression (Fig. S3P). We then generated the stable knockdown of CCNA2 cells by specific short hairpin RNAs (shRNAs) (Fig. 2K). We found that the knockdown of CCNA2 decreased the CRC cell cycles (Supplementary Table 8) and cell proliferation (Fig. 2L). These results suggested that CCNA2 is responsible for the CRC cell cycles and cell proliferation.

Previous reports have shown that the high expression of HMGA2 in CRC predicts a poor prognosis [19]. We performed IHC staining to determine the expression of HMGA2 protein in CRC tissue microarrays (Cohort 2: *n* = 30 and Cohort 3: *n* = 72). IHC staining showed that it was significantly higher in CRC than paired adjacent normal tissues (Fig. S3Q-S3T, Supplementary Tables 9, and Supplementary Table 10). The expression of HMGA2 is also highly expressed in CRC tissues, according to the GEPIA database (Fig. S3U). Furthermore, the expression of HMGA2 protein was positively correlated with 6PGD expression (Fig. S3V). To more carefully elucidate the role of HMGA2 in CRC, we generated the stable knockdown of HMGA2 cells by specific short hairpin RNAs (shRNAs) (Fig. 2M) and found that the knockdown of HMGA2 decreased CRC cell migration (Fig. 2N) and cell invasion (Fig. 2O and Fig. S3W). Taken together, these results suggested that CCNA2 and HMGA2 promote CRC cell proliferation and cell migration, respectively.

### 6PGD non-metabolic activity modulates the expression of CCNA2 and HMGA2 and is dependent on p53

**Fig. 3:**
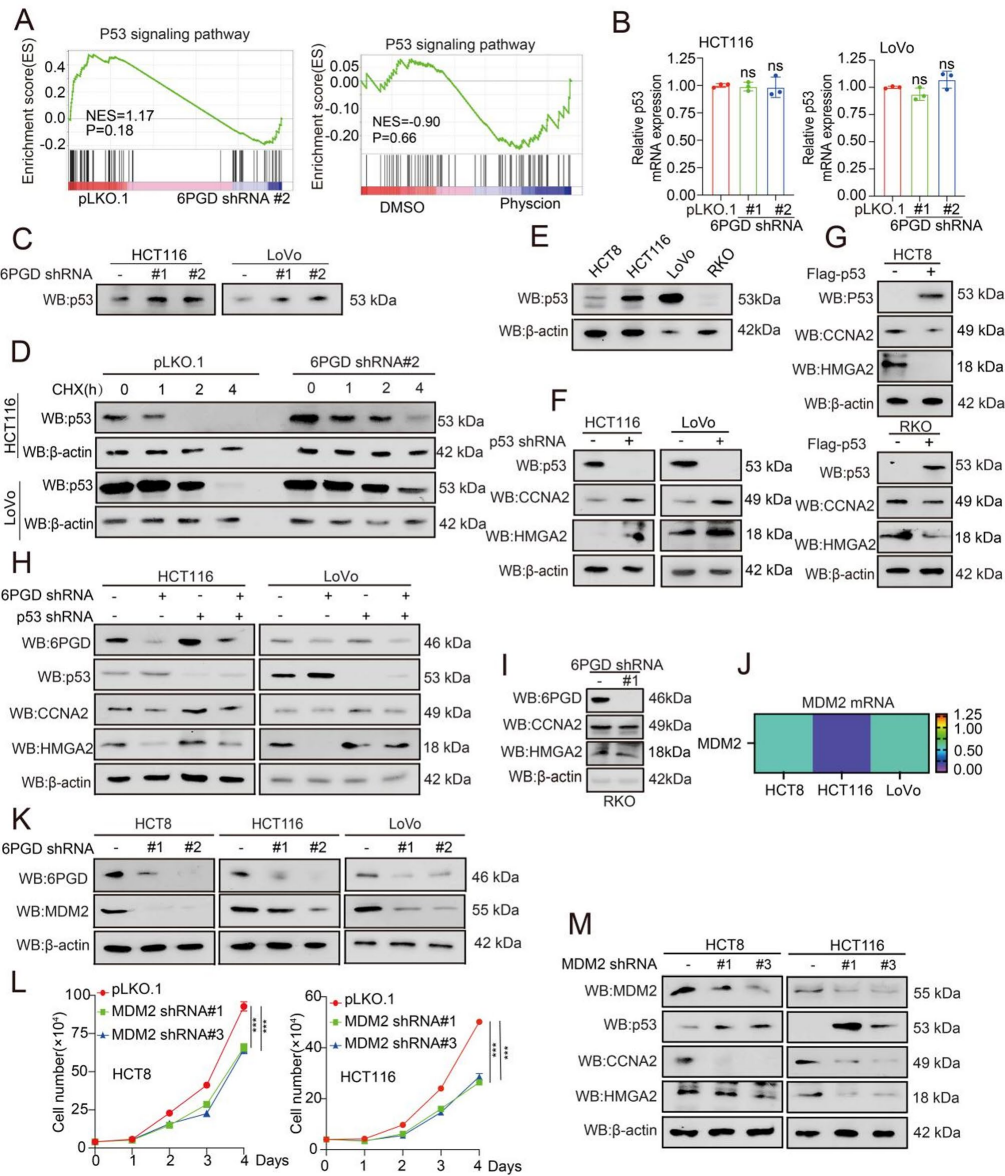
6PGD non-metabolic activity dependent on p53 to modulates the expression of CCNA2 and HMGA2. (**A**) The p53 signaling pathway was enriched in 6PGD related pathways in which RNA-seq were analyzed by gene set enrichment analysis (GSEA). (**B**) The mRNA level of p53 was detected in HCT116 and LoVo cells with stable knockdown of 6PGD by qRT-PCR assays. (**C**) The protein level of p53 was detected in HCT116 and LoVo cells with stable knockdown of 6PGD by western blotting. The actin was same as Fig. 2J. (**D**) The protein stability of p53 was determined in the knockdown of 6PGD or vector control CRC cells treated with or without CHX by western blotting. (**E**) The protein level of p53 was detected in HCT8, HCT116, LoVo, and RKO cells. (**F**) The protein level of CCNA2 and HMGA2 were examined in the knockdown of p53 or vector control CRC cells by western blotting. (**G**) The protein level of CCNA2 and HMGA2 were examined in CRC cells with exogenous expression of p53 by western blotting. (**H**) The protein level of CCNA2 and HMGA2 were examined in the knockdown of 6PGD or vector control CRC cells with or without knockdown of p53 by western blotting. (**I**) The protein level of CCNA2 and HMGA2 were examined in the knockdown of 6PGD RKO cells. (**J**) The mRNA level of MDM2 was determined in the knockdown of 6PGD or vector control CRC cells by qRT-PCR. (**K**) The protein level of MDM2 was determined in the knockdown of 6PGD or vector control CRC cells by western blotting. (**L**) Cell proliferation was determined in the knockdown of MDM2 or vector control CRC cells by cell number counting assay. (**M**) The protein level of p53, CCNA2, and HMGA2 were examined in the knockdown of MDM2 or vector control CRC cells by western blotting. The data represent mean values ± SD from three replicates of each sample (*0.01 < *p* < 0.05; **0.001 < *p* < 0.01; ***p < 0.001)

Subsequently, we determined the precise underlay mechanism by which 6PGD promotes the cell cycles and cell migration by regulating the expression of CCNA2 and HMGA2, respectively. We analyzed transcriptome sequencing (RNA-seq) results and found that the protein levels of 6PGD, as opposed to the enzymatic activity, regulating the p53 signaling pathway based on the GSEA analysis (Fig. 3A). The qRT-PCR and western blot results showed that the knockdown of 6PGD had no effect on p53 mRNA expression but increased the expression of p53 protein (Fig. 3B and 3C). Moreover, Physcion treatment exerted no effect on the expression of p53, while it increased the phosphorylation level of AMPK (Fig. S4A). Our previous studies showed that p-AMPK was regulated by the enzymatic activity of 6PGD. Thus, we wonder whether 6PGD regulates the stability of p53 protein. We found that the knockdown of 6PGD increased the p53 protein stability by the protein stability assay (Fig. 3D).

As p53 regulates various forms of gene expression as a transcription factor, we then explored whether 6PGD regulates the expression of CCNA2 and HMGA2 in CRC cells mediated by p53. Firstly, we generated the stable knockdown of p53 by shRNA, and we found that the knockdown of p53 increased the expression of CCNA2 and HMGA2 in the p53 higher expressed HCT116 and LoVo cells (Fig. 3E and F, and Fig. S4B). In contrast, we found that the exogenous expression of p53 decreased the expression of CCNA2 and HMGA2 in the p53 lower expressed HCT8 and RKO cells (Fig. 3E and 3G and Fig. S4C). Secondly, we suppressed p53 in the knockdown of 6PGD cells and found that the suppression of p53 rescued the decreased expression of CCNA2 and HMGA2 in the knockdown of 6PGD cells (Fig. 3H). Lastly, the knockdown of 6PGD not significantly affected the expression of CCNA2 and HMGA2 proteins in RKO cell (Fig. 3I). We converted express wild-type 6PGD (6PGD WT) or catalytically inactive 6PGD (6PGD K76R) into HCT8, HCT116, and LoVo cells and found that 6PGD-K76R also altered the expression of CCNA2 and HMGA2, which is same as 6PGD WT (Fig. S4D). Consistent with previous observations, the knockdown of 6PGD dramatically reduced the expression of CCNA2 and HMGA2 protein, whereas the exogeneous expression of both 6PGD-WT and 6PGD K76R elevated the expression of CCNA2 and HMGA2 (Fig. S4E). These findings showed that 6PGD regulates the expression of CCNA2 and HMGA2 in CRC cells mediated by p53 in a non-metabolic-dependent manner.

### 6PGD non-metabolic activity regulates p53 protein stability in a manner that is dependent on MDM2

As MDM2 regulates the stabilization of p53 protein, we wondered whether the non-metabolic activity of 6PGD regulates p53 stability mediated by MDM2. Firstly, we found that the knockdown of 6PGD reduced the expression of MDM2 mRNA and protein (Fig. 3J and 3K). Thus, we generated the stable knockdown of MDM2 cells and found that the knockdown of MDM2 decreased HCT8 and HCT116 cells’ proliferation (Fig. 3L). In addition, the knockdown of MDM2 increased p53 expression and decreased CCNA2 and HMGA2 expression (Fig. 3M). However, Physcion treatment exerted no effect on the expression of MDM2 (Fig. S4A). We converted express 6PGD WT or 6PGD-K76R into HCT8, HCT116, and LoVo cells and found that the exogeneous expression of both 6PGD WT and 6PGD K76R elevated the expression of MDM2 (Fig. S4D). Consistent with previous observations, the knockout of 6PGD dramatically reduced the protein level of MDM2, whereas the exogeneous expression of both 6PGD WT and 6PGD K76R elevated its protein levels (Fig. S4E). These results suggested that 6PGD regulates the expression of CCNA2 and HMGA2 through the MDM2-p53 axis in a non-metabolic-dependent manner.

### 6PGD interacts with ALKBH5

**Fig. 4:**
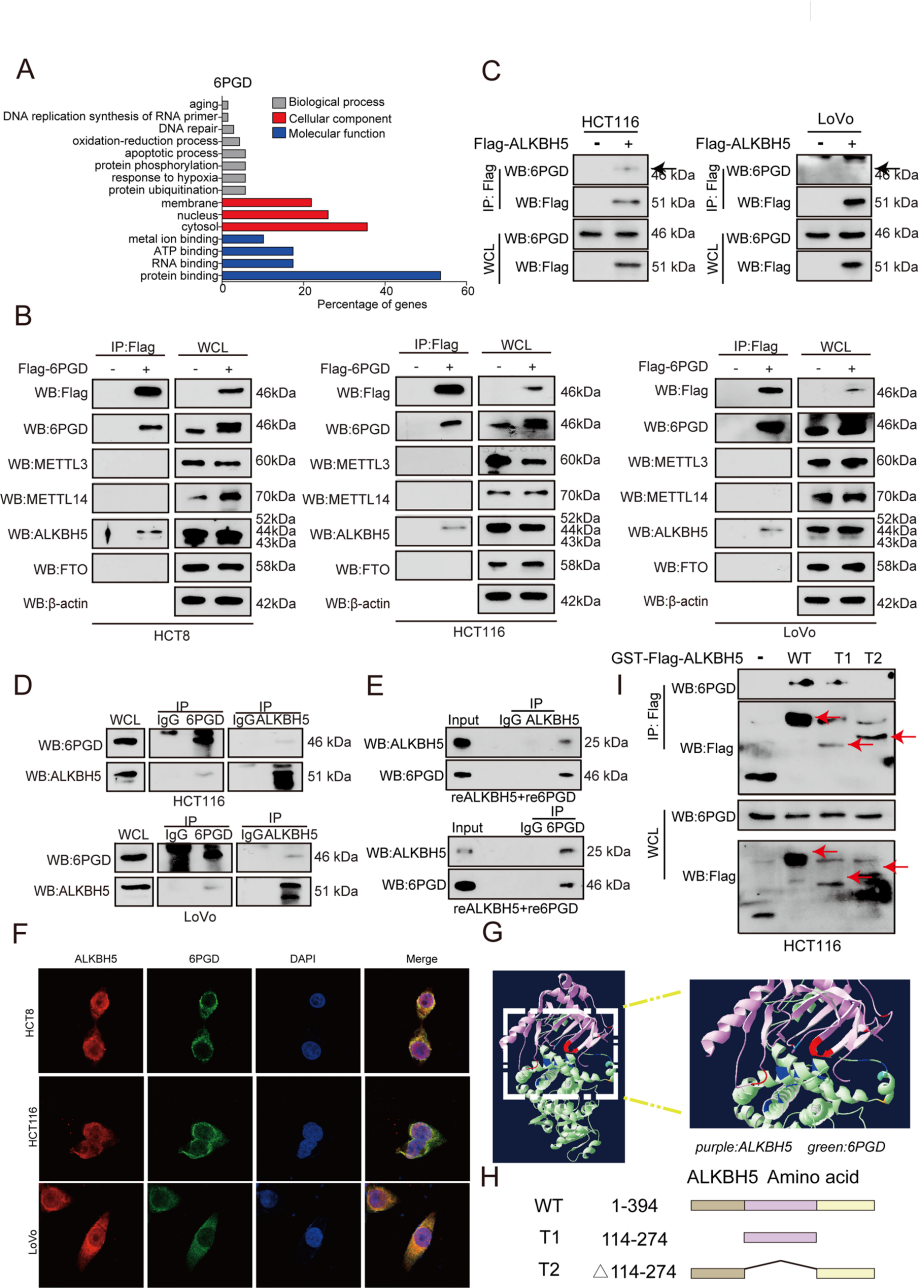
6PGD binds to ALKBH5. (**A**) The target protein bound by 6PGD were analyzed by GO pathway analysis. (**B**) The expression of m^6^A-related protein were determined by Flag-pull down assay in CRC cells by western blotting. (**C**) The interaction between endogenous 6PGD and Flag-ALKBH5 were determined in HCT116 and LoVo cells by in vivo Flag-pull down assay. (**D**) The interaction between endogenous 6PGD and endogenous ALKBH5 were determined in HCT116 and LoVo cells by in vivo Co-IP assay. (**E**) The interaction between recombinant 6PGD and recombinant ALKBH5 were determined by in vitro Co-IP assay. (**F**) Detection of intracellular localization of ALKBH5 (red) and 6PGD (green) in CRC cells by IF. (**G**) Molecular dynamics (MD) simulation picture of the combination of ALKBH5 with 6PGD. Right exploded view shows ALKBH5 (purple), 6PGD (green), and their binding regions (red and blue). (**H**) Schematic diagram showed the structure of ALKBH5 WT (1-394) and truncation mutants: T1 (114–274), and T2 (Δ114–274). (**I**) Flag-tagged ALKBH5 WT or the indicated truncation mutants were overexpressed in HCT116 cells. Extracts were immunoprecipitated with Flag antibody to examine the ALKBH5 WT, the indicated truncation mutants, and 6PGD by western blotting. The data represent mean values ± SD from three replicates of each sample (*0.01 < *p* < 0.05; **0.001 < *p* < 0.01; ***p < 0.001)

To explore the mechanism of 6PGD in regulating MDM2, a Flag pull-down assay was conducted to identify proteins interacting with 6PGD, which was subjected to mass spectrometry (MS) analysis. A gene ontology (GO) analysis was conducted on the protein enriched by 6PGD, which are crucial for RNA binding and protein binding (Fig. 4A). A further analysis of Flag pull-down data revealed that 6PGD binds to ALKBH5 in the RNA binding-related categories (Fig. 4A). The interaction between 6PGD and ALKBH5 proteins (not METTL3, METTL14, and FTO) in HCT116, HCT8, and LoVo cells was confirmed through 6PGD Flag pull-down assays (Fig. 4B). In addition, the interaction between 6PGD and ALKBH5 proteins in HCT116 and LoVo cells was confirmed through ALKBH5 Flag pull-down assays (Fig. 4C). Lastly, an in vivo co-IP assay, in vitro co-IP binding assays, microscale thermophoresis (MST) assays showed that 6PGD directly interacted with ALKBH5 (Fig. 4D and 4E and S5A), and found that there are strong binding affinity (kd = 1.626 × 10–6 mol/L) between ALKBH5 and 6PGD (Fig. S5A). Moreover, immunofluorescence staining showed that 6PGD and ALKBH5 co-localized in the cytoplasm (Fig. 4F). Through an analysis of the results of 6PGD and ALKBH5 using the software Discovery Studio, we found that the amino acid region of ALKBH5’s 114–274 is related to 6PGD interactions (Fig. 4G). To verify this result, we constructed a series of deletion mutants of ALKBH5 (Fig. 4H). The Flag pull-down results showed that 6PGD interacts with ALKBH5 on the amino acid region 114–274 (Fig. 4I). Together, these results suggested that 6PGD interacts with ALKBH5. The above results showed that 6PGD and ALKBH5 bind to each other.

### 6PGD promotes RNA m^6^A modification through interactions with ALKBH5

**Fig. 5:**
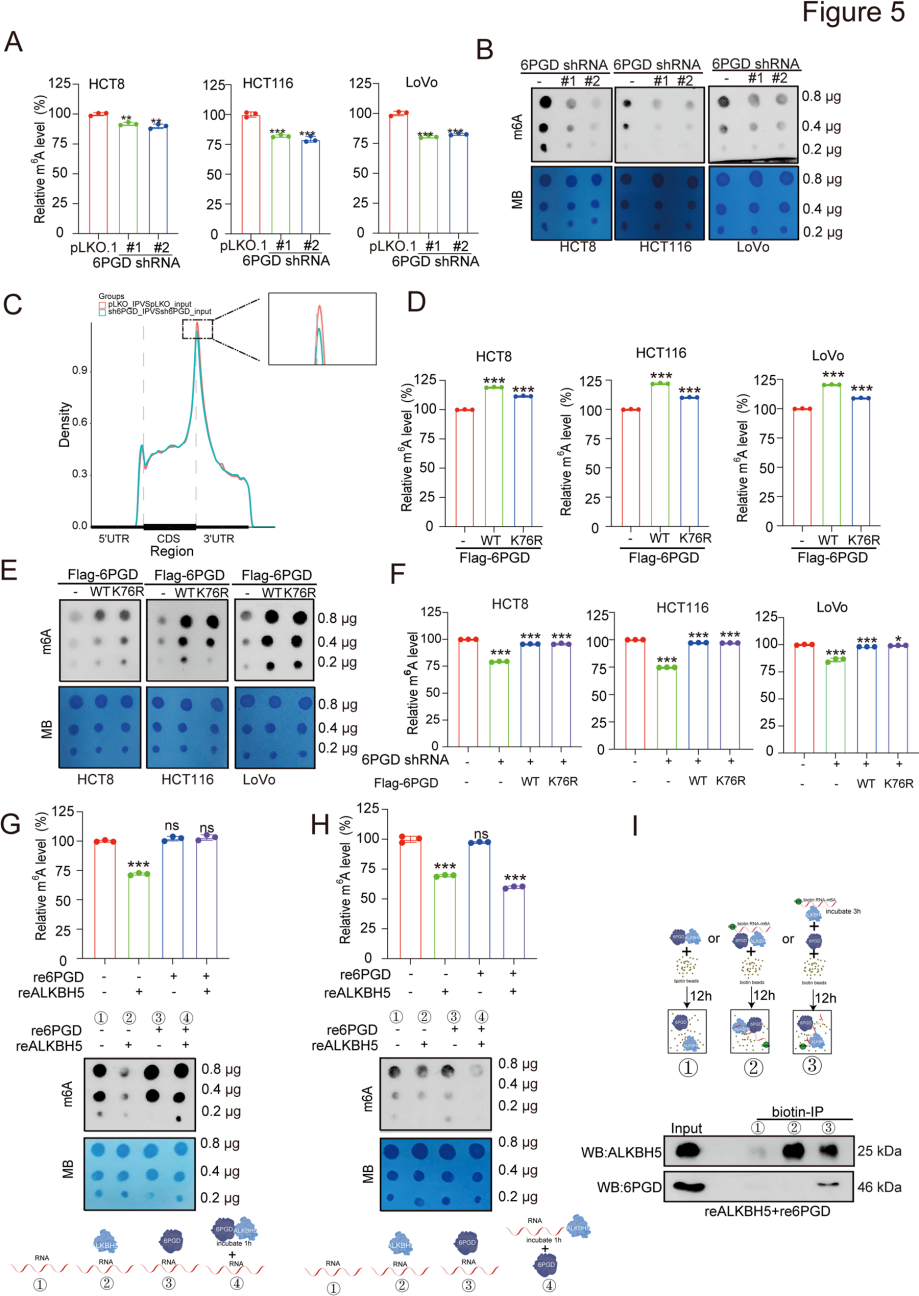
6PGD regulates the m^6^A level in a catalytic-activity-independent manner. (**A**) The m^6^A levels were determined by the m^6^A RNA Methylation Quantification Kit (Colorimetric) in the knockdown of 6PGD or vector control CRC cells. (**B**) The m^6^A levels were determined by dot blotting assay in the knockdown of 6PGD or vector control CRC cells. (**C**) The density distribution of m^6^A peaks in messenger RNA (mRNA) transcripts based on knockdown of 6PGD m6A-seq. (**D**) The m6A levels were determined by the m^6^A RNA Methylation Quantification Kit (Colorimetric) in CRC cells with exogenous expression of 6PGD WT or 6PGD K76R. (**E**) The m^6^A levels were determined by dot blotting assay in CRC cells with exogenous expression of 6PGD WT or 6PGD K76R. (**F**) The m^6^A levels were determined by the m6A RNA Methylation Quantification Kit (Colorimetric) in the knockdown of 6PGD CRC cells with exogenous expression of 6PGD WT or 6PGD K76R. (**G**) The in vitro methylation assay was employed for methyltransferase activity of reALKBH5 pre-incubated with or without re6PGD by the m^6^A RNA Methylation Quantification Kit (Colorimetric) (left) and dot blotting assay (right). (**H**) The in vitro methylation assay was employed for methyltransferase activity of reALKBH5 pre-incubated with RNA from HCT8 cells and followed with re6PGD by the m^6^A RNA Methylation Quantification Kit (Colorimetric) (left) and dot blotting assay (right). (**I**) The in vitro interaction between recombinant ALKBH5 and biotin-RNA were examined by biotin-pull down pre-incubated with or without re6PGD. The data represent mean values ± SD from three replicates of each sample (*0.01 < *p* < 0.05; **0.001 < *p* < 0.01; ***p < 0.001)

Mounting evidence shows that ALKBH5 is involved in mRNA m^6^A modification, suggesting that 6PGD might be involved in m^6^A modification. To determine whether the regulation of 6PGD in relation to mRNA m^6^A modification was mediated by ALKBH5, we first evaluated the functional role of 6PGD on mRNA m^6^A modification in CRC cells. To test this hypothesis, we first knocked down 6PGD in CRC cells and found that the expression of ALKBH5, METTL3, METTL14, and FTO were not significantly changed in CRC cells with the knockdown of 6PGD (Fig. S5B). However, we found that 6PGD knockdown (or knock out) reduced mRNA m^6^A modification in CRC cells using a m^6^A detection kit and dot blotting assay (Fig. 5A and 5B, and Fig. S5C). In addition, the methylated RNA immunoprecipitation sequencing (MeRIP-Seq or m^6^A-Seq) results showed that the knockdown of 6PGD reduced m^6^A levels in the CDS region and 3 ‘UTR region of mRNA compared with control cells (Fig. 5C). However, we found that Physcion treatment did not affect mRNA m^6^A modification in CRC cells (Fig. S5D-S5E). Therefore, we further speculate that the expression of 6PGD, rather than the enzyme activity, affects its binding to ALKBH5 and influences its m^6^A modification. Firstly, we found that the exogeneous expression of both 6PGD WT and 6PGD K76R altered mRNA m^6^A modification in CRC cells (Fig. 5D-5E). In addition, either 6PGD WT or 6PGD K76R exogeneous expression in the knockdown of 6PGD CRC cells counteracted the decrease in the total m^6^A modification levels caused by the knockdown of 6PGD (Fig. 5F and Fig. S5F). Lastly, we found that the in vivo and in vitro binding assays showed that ALKBH5 also interacted with 6PGD K76R (Fig. S5G-S5H). Together, these results suggested that 6PGD serves as an m^6^A regulator in CRC cells independently of its catalytic activity mediated by interacting with ALKBH5.

To explore the underlying mechanisms by which 6PGD exerts its regulatory function on mRNA m^6^A modification in CRC carcinogenesis, we endeavored to further test whether 6PGD binding to ALKBH5 affects the function of ALKBH5 in regulating mRNA m^6^A modification. Firstly, we incubated bacterially expressed recombinant 6PGD (re6PGD) with recombinant ALKBH5 (reALKBH5; aa:66–292) and performed an in vitro methylation assay; when reALKBH5 pre-incubated with re6PGD, the m^6^A modification on mRNA is not removed by ALKBH5 (Fig. 5G). However, when we incubated mRNA with ALKBH5 and then incubated it with re6PGD, re6PGD did not affect the demethylation activity of ALKBH5 (Fig. 5H). These results suggested that the binding of 6PGD to ALKBH5 can affect the demethylation activity of ALKBH5.

We next sought to determine whether the interaction between 6PGD and ALKBH5 was affected by RNA; our in vitro binding assay and MST assays showed that the strong interaction between 6PGD and ALKBH5 was destroyed when the reALKBH5 was pre-incubated with mRNA (Fig. 5I and Fig. S5A), while the 6PGD protein did not bind to mRNA directly, but the ALKBH5 protein binded to mRNA directly (Fig. S5I-S5J). These results clearly showed that 6PGD binded to ALKBH5 and regulated the demethylation activity of ALKBH5.

### 6PGD regulates the m6A level of MDM2 mRNA through ALKBH5

**Fig. 6:**
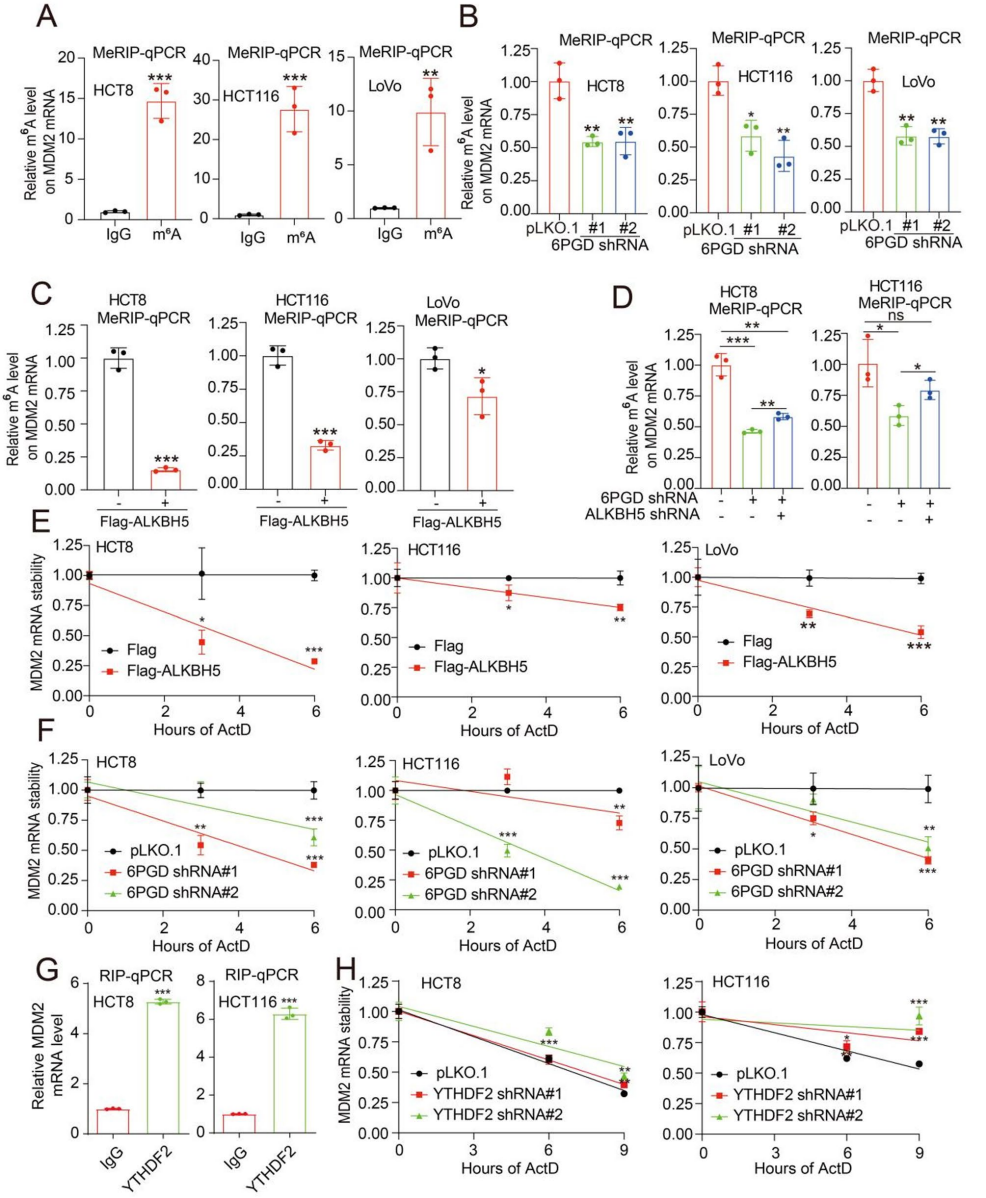
6PGD regulates the m^6^A level on MDM2 mRNA mediated by ALKBH5. (**A**) The MeRIP-qPCR analysis of MDM2 m^6^A levels in CRC cells. (**B**) The MeRIP-qPCR analysis of MDM2 m^6^A levels in knockdown of 6PGD cells and control CRC cells. (**C**) The MeRIP-qPCR analysis of MDM2 m^6^A levels in CRC cells with exogenous expression of ALKBH5. (**D**) The MeRIP-qPCR analysis of MDM2 m^6^A levels in knockdown of 6PGD cells and control CRC cells with or without knockdown ALKBH5. (**E**) The qPCR analysis for the mRNA stability of MDM2 in CRC cells with exogenous expression of ALKBH5. (**F**) The qPCR analysis for the mRNA stability of MDM2 in the knockdown of 6PGD and control CRC cells. (**G**) RIP-qPCR analysis to examine the interaction between MDM2 mRNA and YTHDF2. (**H**) The qPCR analysis for the mRNA stability of MDM2 in CRC cells with knockdown of YTHDF2. The data represent mean values ± SD from three replicates of each sample (*0.01 < *p* < 0.05; **0.001 < *p* < 0.01; ***p < 0.001)

In this study, we found that 6PGD regulates m^6^A modification in CRC. Thus, we explored whether 6PGD regulates MDM2 mRNA mediated by m^6^A modification. Firstly, we applied the MeRIP-qPCR assay and found that there was m^6^A modification on MDM2 mRNA in CRC cells (Fig. 6A). We then analyzed MeRIP-Seq data and found that m^6^A modification was decreased on MDM2 mRNA with the knockdown of 6PGD (Fig. S6A). In addition, we confirmed that the knockdown of 6PGD reduced the m^6^A modification on MDM2 mRNA via a MeRIP-qPCR assay (Fig. 6B). However, 6PGD did not significantly affect the m^6^A modification on CCNA2 and HMGA2 mRNA (Fig. S6B-S6C). Consistent with previous observations, either the exogenous expression of 6PGD WT or 6PGD K76R in the knockdown of 6PGD CRC cells could remedy the decreased m^6^A modification on MDM2 mRNA, which was caused by the knockdown of 6PGD (Fig. S6D). These results suggested that 6PGD regulates the m^6^A modification on MDM2 mRNA in a non-metabolic-dependent manner.

In this study, we found that 6PGD interacts with ALKBH5. Thus, we wondered whether 6PGD regulates m6A modification on MDM2 mRNA mediated by ALKBH5. We subsequently explored whether 6PGD regulates m6A modification on MDM2 mediated by ALKBH5. Firstly, we performed MeRIP-Seq to determine the global effect of ALKHB5 on m6A modification in the knockdown ALKBH5 cells. The MeRIP-Seq results showed that the knockdown of ALKHB5 induced m6A levels in the CDS region and 3 ‘UTR region of mRNA compared with control cells (Fig. S6E). We then analyzed MeRIP-Seq data and found that m6A modification was increased on MDM2 mRNA with the knockdown of ALKHB5 (Fig. S6F). In addition, we confirmed that m6A modification on MDM2 mRNA was decreased in the CRC cells with the exogeneous expression of ALKBH5 was analyzed by MeRIP-qPCR assays (Fig. 6C); this induced the decreased expression of MDM2, HMGA2, and CCNA2, while increasing the expression of p53 (Fig. S6G), as well as the protein stability of p53 (Fig. S6H). We found that the knockdown of ALKBH5 counteracted the decreased m6A modification on MDM2 mRNA in the knockdown of 6PGD cells (Fig. 6D) and remedied the decreased MDM2, HMGA2, and CCNA2 expression and the increased p53 expression (Fig. S6I). Finally, we found that the stability of MDM2 mRNA was decreased in cells with the exogenous expression of ALKBH5 (Fig. 6E). We also found that the stability of MDM2 mRNA was reduced upon the knockdown of 6PGD (Fig. 6F).

As the m^6^A modification is recognized and bound by m^6^A-binding proteins (Readers), such as, YTHDF2, which mediates the degradation of mRNA. Thus, we wonder whether the MDM2 mRNA stability was regulated by YTHDF2. Next, we performed a RNA immunoprecipitation qPCR (RIP-qPCR) assays to explore the direct interaction between the YTHDF2 and MDM2 mRNA. RIP-qPCR assays showed that YTHDF2 bound to MDM2 mRNA (Fig. 6G). Moreover, the stability of MDM2 mRNA was increased in cells with the knockdown of YTHDF2 (Fig. 6H). Finally, the levels of MDM2 mRNA and protein were also markedly increased upon YTHDF2 knockdown in CRC cells (Fig. S6J-S6K). Taken together, these findings showed that 6PGD modulates the m^6^A modification on MDM2 mRNA mediated by ALKBH5 and regulates the stability of MDM2 mRNA mediated by YTHDF2.

We also determined the biological functions of MDM2 in CRC using the same CRC tumor tissue microarrays and confirmed the MDM2 expression in clinical samples via IHC staining (Cohort 2: *n* = 30). According to the intensity and area of MDM2 staining, MDM2 was significantly higher in CRC compared to paired adjacent normal tissues (Fig. S6L-S6M, Supplementary Tables 11-Table 12).

### The suppression of 6PGD expression enhances chemotherapy sensitivity

**Fig. 7:**
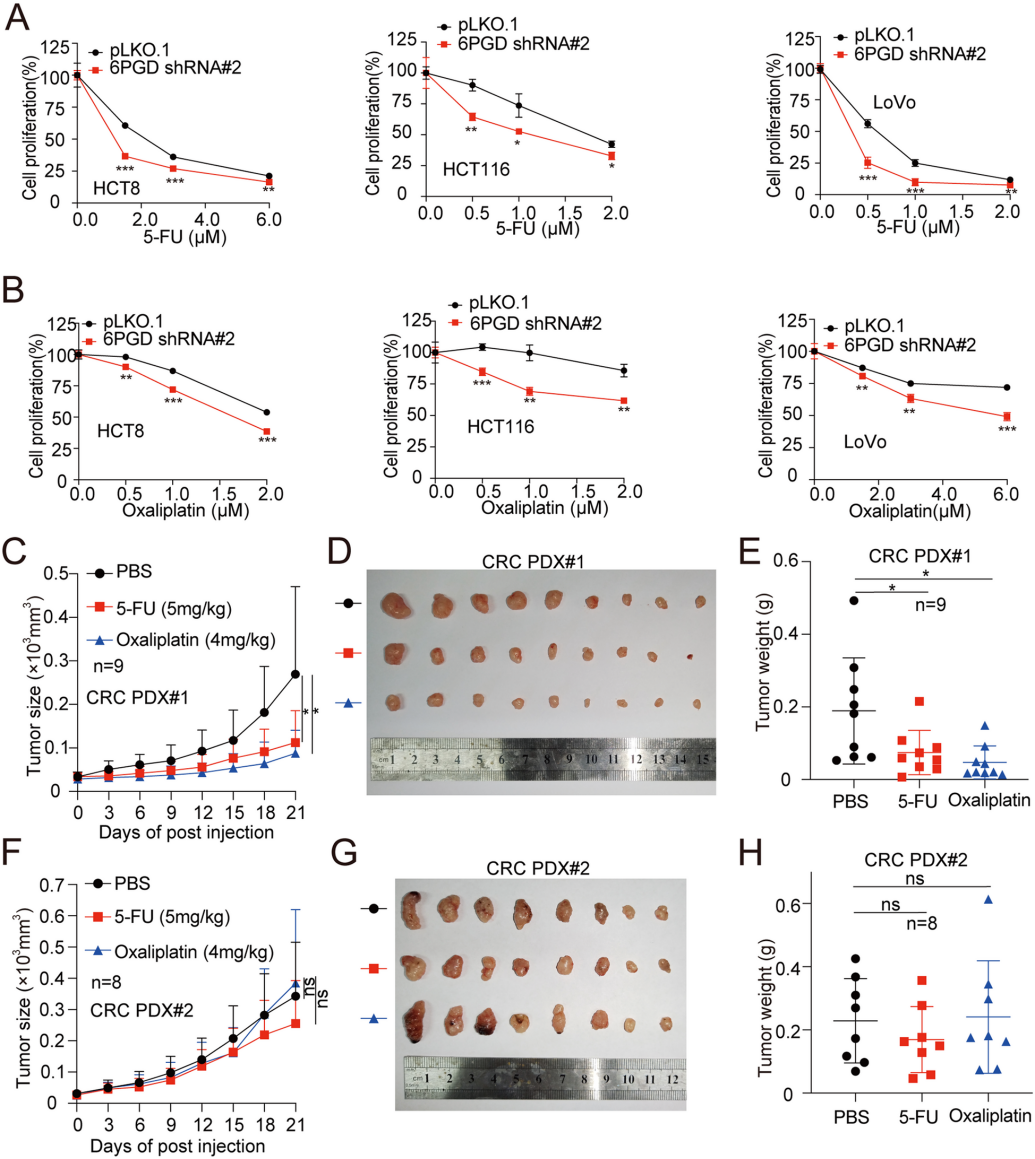
Supression of 6PGD expression can enhance chemotherapy sensitivity. (**A**) Cell proliferation was determined by cell number counting assay in CRC cells with stable knockdown of 6PGD when treated with or without the indicated dose of 5-FU. (**B**) Cell proliferation was determined by cell number counting assay in CRC cells with stable knockdown of 6PGD when treated with or without the indicated dose of Oxaliplatin. (**C**) Tumor growth was compared between xenograft nude mice bearing with CRC PDX #1 injection with 5-FU or Oxaliplatin (*n* = 9). (**D**) All tumors from nude mice were shown. (**E**) Tumor mass in xenograft nude mice injected with PDX#1 tumor and treated with 5-FU or Oxaliplatin (*n* = 9). (**F**) Tumor growth was compared between xenograft nude mice bearing with CRC PDX #2 injection with 5-FU or Oxaliplatin (*n* = 8). (**G**) All tumors from nude mice were shown. (**H**) Tumor mass in xenograft nude mice injected with PDX#2 tumor and treated with 5-FU or Oxaliplatin (*n* = 8). The data represent mean values ± SD from three replicates of each sample (*0.01 < *p* < 0.05; **0.001 < *p* < 0.01; ***p < 0.001)

Resistance to chemotherapy drugs, including 5-FU and oxaliplatin, which commonly occurs during the treatment of CRC. To further determine the therapeutic benefit of targeting 6PGD in combination with chemotherapy, we tested the sensitivity of chemotherapy in 6PGD knockdown cells. We found that the knockdown of endogenous 6PGD significantly enhanced the sensitivity to chemotherapy treatments, including 5-FU and oxaliplatin (Fig. 7A and 7B, Fig. S7A), while the targeting of 6PGD by Physicion does not significantly increase the sensitivity of CRC cells to chemotherapeutic drugs (Fig. S7B). Taken together, these data suggested that altering the protein levels but not the enzyme activity of 6PGD significantly enhances chemotherapy sensitivity in CRC.

To further explore the role of 6PGD in regulating chemotherapy sensitivity, we successfully constructed two CRC PDX and found that the expression of 6PGD protein in PDX#1 was lower than that of PDX#2 (Fig. S7C). Thus, we wondered whether the expression of 6PGD protein in CRC PDX models regulates chemotherapy sensitivity. We then detected the anti-tumor sensitivity of 5-FU and oxaliplatin, two commonly used chemotherapy drugs for CRC, in these two PDX models. We found that 5-FU and oxaliplatin significantly decreased tumor growth (Fig. 7C and 7D) and tumor masses (Fig. 7E) compared with mice receiving PBS in PDX#1 with the lower expression of 6PGD, while not affecting the tumor growth rate (Fig. 7F and 7G) and tumor masses (Fig. 7H) in PDX#2, with the higher expression of 6PGD protein. Neither 5-FU nor oxaliplatin exerted significant effects on the body weight of mice (Fig. S7D-S7E). Taken together, these data suggested that the non-metabolic activity of 6PGD plays a key role in enhancing the anti-tumor effect of 5-FU and oxaliplatin in CRC.

## Discussion

6PGD is known as a key enzyme in PPP flux, which is involved in metabolizing 6-phosphogluconate (6-PG) to ribulose-5-phosphate (Ru-5-P), and emerging evidence has revealed that the up-regulation of 6PGD expression or enzyme activity serves as a contributor to lung cancer, thyroid cancer, colon cancer, head and neck cancer, and ovarian cancer. While the non-metabolic functions of 6PGD in cancer progression have not been explored, it remains poorly understood whether more non-metabolic functions of 6PGD are related to cancer and other diseases. Here, we demonstrated that the non-metabolic activity of 6PGD regulates p53 protein stability to promote CRC tumor growth and migration through the ALKBH5-MDM2-p53-CCNA2/HMGA2 axis. The non-metabolic function of 6PGD may provide additional advantages related to the fitness of CRC cells. Thus, our findings amplify the effects of cancer drivers in promoting tumor progression, including CRC.

Employing a Flag pull-down assay and mass spectrometry (MS) analysis, we uncovered protein enriched by 6PGD, which are crucial for RNA binding, including ALKBH5, is a key demethylase to remove m^6^A modification on mRNA [20]. m^6^A is the most common internal RNA modification in the consensus sequence of 5’-RRACH-3‘ [21]. Firstly, we confirmed the interaction between ALKBH5 and 6PGD in vitro and in vivo to explore the effect of 6PGD on its binding to ALKBH5. Secondly, we employed MeRIP-Seq and showed that the knockdown of 6PGD reduced m^6^A modification in the CDS region and 3 ‘UTR region on mRNA. Conversely, the 6PGD inhibitor treatment does not have an effect on m^6^A modification. To a certain extent, this indicates that the function of 6PGD in relation to m^6^A modification is independent of enzyme activity.

To explore how the non-metabolic activity of 6PGD regulates CRC tumor growth and migration, we performed RNA-seq based on the knockdown of 6PGD, 6PGD inhibitor treatment, 6PGD wildtype and 6PGD K76R samples, and found that 6PGD serves a function in the cell cycles and migration that is independent of enzyme activity. We demonstrated that the knockdown of 6PGD prevented the CRC cells from S phase entry into M phase. Cyclins and cyclin-dependent kinases (CDKs) are key regulators of the cell cycles [22]. CCNA2/CDK2 promotes S phase progression and regulates G2 entry. Indeed, we found that the knockdown of 6PGD, not the inhibitor of it decreased the expression of CCNA2. We also demonstrated that the knockdown of 6PGD inhibits CRC migration. HMGA2 is a member of the non-histone chromosomal high mobility group (HMG) protein family, which participates in several biological processes, including metastasis [23]. Indeed, we found that the knockdown of 6PGD, not the inhibitor of it decreased the expression of HMGA2. Thus, we discovered the moonlighting function of 6PGD in promoting tumor growth and migration via CCNA2 and HMGA2, respectively. p53 has been widely studied for its roles in the cell cycles, cell survival, cell migration, DNA damage repair, and apoptosis [24]. To explore how 6PGD regulates CCNA2 and HMGA2, respectively, we examined whether 6PGD regulates CCNA2 and HMGA2 expression mediated by p53. Indeed, the decreased expression of CCNA2 and HMGA2 in the knockdown of 6PGD cells was counteracted by knocking down p53. Finally, we extended our findings to patients with CRC and found that CCNA2 or HMGA2 was remarkably upregulated in the CRC and uncovered a correlation with the expression of 6PGD.

**Fig. 8:**
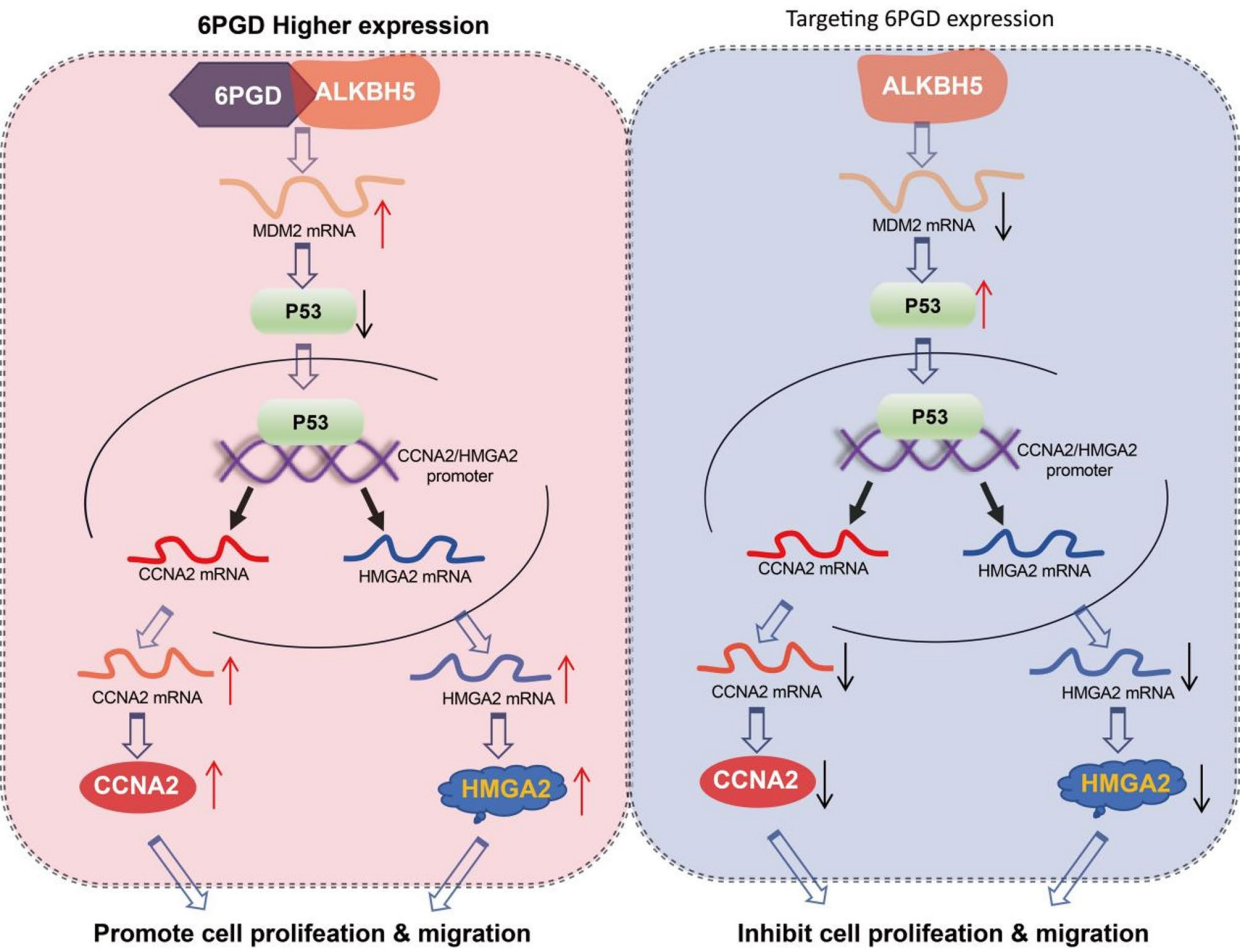
Proposed model: the non-metabolic function of 6PGD coordinates CCNA2 and HMGA2 expression to drive colorectal cancer progression and drug response.

MDM2 is a negative regulator of the tumor suppressor p53; it regulates the stability of p53 protein and is often strongly expressed in cancers [25]. We demonstrated that 6PGD directly binds to the m^6^A demethyltransferase ALKBH5 and inhibits the activity of ALKBH5 to remove m^6^A modification on MDM2 mRNA, leading to the regulation of the stability of MDM2 mRNA mediated by YTHDF2 reader. Indeed, we found that MDM2 was remarkably up-regulated in the CRC. In conclusion, we identified the moonlighting function of 6PGD, which serves non-metabolic roles in promoting the cell cycles and migration in CRC. Mechanically, 6PGD binds to the ALKBH5 and then inhibits the activity of ALKBH5 to remove m^6^A modifications on mRNA through its moonlighting function of protein-protein interactions to activate the downstream p53-signaling pathway mediated by regulating MDM2 mRNA stability. Meanwhile, p53 drives the cell cycles and migration by regulating the expression of CCNA2 and HMGA2 (Fig. 8). Our findings support the notion that non-classical metabolic functions of 6PGD have a potential therapeutic value of killing CRC, beyond only targeting its metabolic activity.

Chemotherapy is one of the most common and effective treatments for CRC. However, because many patients acquire drug resistance, chemotherapy has extensive limitations. Therefore, we further investigated whether targeting 6PGD can enhance the anti-tumor effect of chemotherapy drugs in CRC. Endogenous 6PGD gene knockdown can significantly enhance the sensitivity of CRC to most chemotherapy drugs, including 5-FU and oxaliplatin. In contrast, Physcion, an inhibitor of 6PGD, cannot significantly enhance the effect of chemotherapy drugs compared to the knockdown of 6PGD, which also indicates that the non-metabolic activity of 6PGD serves a function in increasing the effect of chemotherapy drugs. In addition, we further demonstrated in vivo that the effects of 5-FU and oxaliplatin were more significant in PDX models with the reduced expression of 6PGD. It is suggested that the non-metabolic activity of 6PGD enhances the sensitivity of CRC to chemotherapy. Although we found that 6PGD-low vs. 6PGD-high expressing PDX models show different sensitivity to chemotherapy. However, we cannot exclude out that PDX#2 is a chemoresistant clone. While, in our work, we just want to mimic and validated the in vitro results that down-regulation of 6PGD expression enhances chemotherapy sensitivity in CRC cells. Thus, this also provides a new direction for the clinical treatment of CRC by targeting non-metabolic roles of 6PGD.

## Conclusion

In conclusion, we demonstrated that 6PGD, the key enzyme in PPP, was remarkably up-regulated in the CRC. Targeting 6PGD dramatically suppressed the cell proliferation and migration of CRC, particularly enhancing the anti-tumor effect of 5-FU and oxaliplatin in CRC in a non-metabolic-functions-dependent manner. This work provides new evidence for 6PGD as a partner that binds to the ALKBH5 and serves a non-metabolic function of regulating the MDM2-p53-CCNA2/ HMGA2 axis. Thus, these findings give a new theoretical basis and scientific significance for exploring the non-classical functions of metabolic enzymes. They also provide a new target for the treatment of CRC.

## Electronic supplementary material

Below is the link to the electronic supplementary material.

Supplementary Material 1

## Data Availability

Further information and requests for resources and reagents should be directed to and will be fulfilled by the Lead Contact, Changliang Shan (changliangshan@nankai.edu.cn ).Raw sequencing data have been deposited at the NCBI Sequence Read Archive and are publicly accessible. Other origin data, including origin image of western blot about this paper, have been deposited at Mendeley and are publicly available. Accession numbers or DOIs are listed in the key resources table.

## Abbreviations

6PGD: 6-phosphogluconate dehydrogenase
CCNA2: Cyclin A2
CRC: Colorectal cancer
HMGA2: High-mobility group AT-hook 2
MDM2: Mouse double minute 2
PDO: Patient-derived organoid
PDX: Patient-derived xenograft
PPP: Pentose phosphate pathway
