# Supplementary material for: The non-metabolic function of 6PGD coordinates CCNA2 and HMGA2 expression to drive colorectal cancer progression and drug response
Source: J Exp Clin Cancer Res. 2025 Jul 3;44:186. doi: 10.1186/s13046-025-03450-3 (PMC12224427; doi:10.1186/s13046-025-03450-3)
Supplement: Supplementary file 1 — Supplementary Material 1 [file 13046_2025_3450_MOESM1_ESM.docx]

**Supplemental information**

**The non-metabolic function of 6PGD coordinates CCNA2 and HMGA2 expression to drive colorectal cancer progression and drug response**

**STAR☆METHODS**

**KEY RESOURCES TABLE**

| **REAGENT or RESOURCE** | | **SOURCE** | **IDENTIFIER** |
| --- | --- | --- | --- |
| **Antibodies** | |  |  |
| 6PGD Rabbit PolyAb | | proteintech | [14718-1-AP](http://www.ptgcn.com/products/PGD-Antibody-14718-1-AP.htm) |
| 6PGD Mouse McAb | | proteintech | 67916-1-Ig |
| MDM2 Mouse McAb | | proteintech | 66511-1-Ig |
| CCNA2 Rabbit PolyAb | | proteintech | 18202-1-AP |
| HMGA2 Rabbit PolyAb | | proteintech | 20795-1-AP |
| ALKBH5 Rabbit PolyAb | | proteintech | 16837-1-AP |
| Flag Rabbit PolyAb | | proteintech | 20543-1-AP |
| p53 Rabbit PolyAb | | proteintech | 10442-1-AP |
| Beta Actin Mouse McAb | | proteintech | 66009-1-Ig |
| Ki67 Rabbit PolyAb | | CST | 9027S |
| METTL3 Rabbit PolyAb | | proteintech | 15073-1-AP |
| METTL14 Rabbit PolyAb | | proteintech | 26158-1-AP |
| FTO Rabbit PolyAb | | proteintech | 27226-1-AP |
| Goat anti-rabbit IgG-HRP | | Solarbio | SE134 |
| Goat anti-mouse IgG-HRP | | Solarbio | SE131 |
| Alexa Fluor 555 Donkey anti-ribbit IgG（H+L) | | Beyotime | A0453 |
| Alexa Fluor 488 Goat anti-mouse IgG（H+L) | | Beyotime | A0428 |
| Goat anti-rabbit IgG（H+L) HRP | | Bioworld | BS13278 |
| Goat anti-mouse IgG（H+L) HRP | | Bioworld | ZJ2020-M |
| **Bacterial and Virus Strains** |  | |  |
| *Trans10 Chemically Competent Cell* | [TransGen Biotech](http://www.baidu.com/link?url=2lik0ADTVVBIG_Pn4V7hg4i7OGiZ9dMUqQ2Jzk3lPlFwz-SOqDvqkcZ8TmHxXHuhSZDDcbrhBCMMD-0RfRDnCq) | | Cat# CD101-01 |
| *Trans* BL21(DE3) Chemically Competent Cell | [TransGen Biotech](http://www.baidu.com/link?url=2lik0ADTVVBIG_Pn4V7hg4i7OGiZ9dMUqQ2Jzk3lPlFwz-SOqDvqkcZ8TmHxXHuhSZDDcbrhBCMMD-0RfRDnCq) | | Cat# CD601-03 |
| **Chemicals, Peptides, and Recombinant Proteins** | | | |
| Physcion | Solarbio | | SP8250 |
| 5-FU | Sigma | | F6627 |
| Oxaliplatin | HENGRUI | | H20000337 |
| Cisplatin | MedChemExpress | | HY-17394 |
| Olaparib | MCE | | HY-10162 |
| Campathecin | MCE | | HY-16560 |
| Etoposide | MCE | | HY-13629 |
| α-KG | Sigma | | 75890 |
| L-ascorbic acid | Sigma | | A92902 |
| 3 × Flag peptide | APExBIO | | A6001 |
| protein A/G sepharose | GE Healthcare Life Sciences | | 17-0618-01 |
| Anti-Flag agarose affinity gel | Sigma-Aldrich | | A4596 |
| Polyethylenimine (PEI) | Polysciences | | 23966 |
| Polybrene | Sigma | | H9268 |
| puromycin | InvivoGen | | Ant-pr-1 |
| TRIzol | Thermo Fisher Scientific | | 15596018 |
| Trypsin 0.5%EDTA | Thermo Fisher Scientific | | 25200-072 |
| Immobilon Western Chemiluminescent HRP Substrate | Millipore | | WBKLS0500 |
| FBS | Excel | | FSP500 |
| RPMI 1640 Medium | Thermo Fisher Scientific | | C11875500BT |
| DMEM Medium | Thermo Fisher Scientific | | C11995500BT |
| RPMI 1640 Medium,no Phenol Red | Thermo Fisher Scientific | | 11835-030 |
| **Critical Commercial Assays** | | | |
| TB Green® Premix Ex TaqTMⅡ(Tli RNaseH plus) | TaKaRa | | RR820A |
| TransStart FastPfu DNA Polymerase | [TransGen Biotech](http://www.baidu.com/link?url=2lik0ADTVVBIG_Pn4V7hg4i7OGiZ9dMUqQ2Jzk3lPlFwz-SOqDvqkcZ8TmHxXHuhSZDDcbrhBCMMD-0RfRDnCq) | | AP221-12 |
| Fast mutagenesis system | [TransGen Biotech](http://www.baidu.com/link?url=2lik0ADTVVBIG_Pn4V7hg4i7OGiZ9dMUqQ2Jzk3lPlFwz-SOqDvqkcZ8TmHxXHuhSZDDcbrhBCMMD-0RfRDnCq) | | FM111-01 |
| Endo-free plasmid maxi kit | Omega | | D6926-03 |
| EasyPure plsmid miniprep kit | [TransGen Biotech](http://www.baidu.com/link?url=2lik0ADTVVBIG_Pn4V7hg4i7OGiZ9dMUqQ2Jzk3lPlFwz-SOqDvqkcZ8TmHxXHuhSZDDcbrhBCMMD-0RfRDnCq) | | EM101-02 |
| EpiQuik m^6^A RNA Methylation Quantification Kit | Gepigentek | | P-9005-96 |
| **Experimental Models: Cell Lines** | | | |
| HEK293T | This paper | | N/A |
| HCT116 | This paper | | N/A |
| HCT8 | This paper | | N/A |
| HT29 | This paper | | N/A |
| LoVo | This paper | | N/A |
| RKO | This paper | | N/A |
| SW480 | This paper | | N/A |
| SW620 | This paper | | N/A |
| NCM460 | This paper | | N/A |
| **shRNA** | | | |
| pLKO.1-puro | TranSheepBio | | N/A |
| pLKO.1-puro-6PGD#1-#2 | TranSheepBio | | N/A |
| pLKO.1-puro-MDM2#1-#3 | TranSheepBio | | N/A |
| pLKO.1-puro-p53 | TranSheepBio | | N/A |
| pLKO.1-puro-HMGA2#1-#2 | TranSheepBio | | N/A |
| pLKO.1-puro-CCNA2 | TranSheepBio | | N/A |
| pLKO.1-puro-ALKBH5#1-#2 | TranSheepBio | | N/A |
| **Oligonucleotides** | | | |
| Primer (qPCR) HMGA2-F | 5'-ACCCAGGGGAAGACCCAAA-3' | | N/A |
| Primer (qPCR) HMGA2-R | 5′-CCTCTTGGCCGTTTTTCTCCA-3′ | | N/A |
| Primer (qPCR) CCNA2-F | 5′-CGCTGGCGGTACTGAAGTC-3′ | | N/A |
| Primer (qPCR) CCNA2-R | 5′-GAGGAACGGTGACATGCTCAT-3′ | | N/A |
| Primer (qPCR) MDM2-F | 5'-GAATCATCGGACTCAGGTACATC-3' | | N/A |
| Primer (qPCR) MDM2-R | 5′-TCTGTCTCACTAATTGCTCTCCT-3′ | | N/A |
| **Recombinant DNA** | | | |
| pCDNA3.1-Flag-6PGD (RNAi resistant) | This paper | | N/A |
| pCDNA3.1-Flag-6PGD K76R (RNAi resistant) | This paper | | N/A |
| pLVX3-ALKBH5 | This paper | | N/A |
| pLVX3-GST-ALKBH5 WT | This paper | | N/A |
| pLVX3-GST-ALKBH5 114-274 | This paper | | N/A |
| pLVX3-GST-ALKBH5 Δ114-274 | This paper | | N/A |
| pRK5-p53 | This paper | | N/A |
| pETM3C-6PGD | This paper | | N/A |
| pETM3C-6PGD K76R | This paper | | N/A |
| pET28a-ALKBH5(66-292) | This paper | | N/A |
| **Deposited data** | | | |
| HCT116-sh6PGD RNA-seq Raw data | This paper | | NCBI：BioProject: PRJNA1142878 |
| HCT116-Physcion RNA-seq Raw data | This paper | | NCBI：BioProject: PRJNA1141723 |
| HCT116-sh6PGD m6A RNA-seq Raw data | This paper | | NCBI：BioProject: PRJNA1140533 |
| **Software and Algorithms** | | | |
| GraphPad Prism 8 | Graph- Pad | | Graph- Pad |
| Adobe Illustrator | Adobe | | Adobe |
| ImageJ | National Institutes of Health | | National Institutes of Health |
| Leica Application Suite X -2.0.1 | Leica Microsystems | | N/A |
| FlowJo-10 | FlowJo | | N/A |
| Compusyn | Chou TC.2010 | | N/A |

**Materials and methods**

**ALKBH5 activity assay**

ALKBH5 activity was carried out in reaction buffer containing 28.3 mM (NH_4_)_2_Fe(SO_4_)_2_·6H_2_O, 3 mM α-KG, 20 mM L-ascorbic acid, 50 mM Hepes, 0.5 mg/mL BSA, 1 μg of recombinant protein ALKBH5 was added and the total volum was 50 μL and incubated at 30°C for 180 minutes. Then dot blotting assay and m^6^A detection kit were used to detect the level of m^6^A, which was used to reflect the activity of ALKBH5.

**m^6^A dot blotting assay**

Dot blotting assay was performed to determine the global m^6^A abundance of total RNA or mRNA. The mRNA was extracted by trizol in CRC cells. In brief, total RNA or mRNA was mixed with SSC buffer and denatured at 65°C for 5 minutes. Then, the RNA samples were loaded on the Amersham Hybond-N+ membrane (RPN119B, GE Healthcare), and crosslinked to the membrane by UV. The membrane was stained by methylene blue for control. Then, the membrane was blocked with 5% skim milk and incubated with m^6^A antibody overnight at 4°C. After rinsed with PBST, the membrane was incubated with secondary antibody for 1 hour. The signal was detected by Immobilon Western HRP Kit (Millipore, USA).

**m^6^A methylated RNA immunoprecipitation-qPCR (MeRIP-qPCR)**

Total RNA was extracted and an equal amount of RNA incubated with m^6^A antibody or normal rabbit IgG mixed Protein A/G Beads (Santa Cruz, CA) in 500 μL buffer containing 40U RNase inhibitors overnight at 4°C. Immune complexes were extensively washed for three times incubated with proteinase K digestion buffer. RNA was finally purified by Trizol/chloroform extraction and analyzed by RT-qPCR. The qPCR primers was listed in supplementary Table 1.

**shRNA-mediated gene silencing and Viral infection**

The short hairpin RNA (shRNA) plasmids targeting 6PGD, MDM2, p53, HMGA2 and CCNA2 were purchased from Transheep Biological Corporation. To establish stable knockdown cells, the HEK293T cells were transfected with lentiviral shRNA constructs plus with viral packaging plasmids (psPAX2 and pMD2.G). The viral supernatant was collected and filtered through 0.45-μm filter after 3 days transfection. Then the HCT8, HCT116 and LoVo cells were transduced by lentivirus and selected with 2 μg/mL puromycin. The knockdown efficacity was determined by western blot analysis.

**Plasmids Cloned and transfections**

Human 6PGD cDNA was cloned into pCDNA3.1 for Flag-tag at N-terminus. 6PGD K76R was cloned into pcDNA3.1 for Flag-tag at N-terminus were performed using the Fast Mutagenesis System (TRAN, China). Human p53 cDNA was cloned into pRK5 for Flag-tag at N-terminus. Human ALKBH5 cDNA was cloned into pLVX3 for Flag-tag at N-terminus. ALKBH5 WT, ALKBH5 114-274, and ALKBH5 Δ114-274 mutations were cloned into pLVX3 for GST-tag and Flag-tag at N-terminus were performed using the Fast Mutagenesis System (TRAN, China). Human 6PGD cDNA was cloned into pETM3C for Flag-tag at N-terminus. 6PGD K76R was cloned into pETM3C for Flag-tag at N-terminus were performed using the Fast Mutagenesis System (TRAN, China). For transient transfections, cells were grown to 60% confluency and transfected with plasmids using polyethylenimine (PEI) Transfection Reagent (Polysciences, US) according to the manufacturer’s protocol.

**Quantitation of mRNA expression by real-time qPCR**

Cells were washed with PBS and total RNA isolated with the TRIzol reagent, then was subjected to reverse transcription using the Hifair® III 1st Strand cDNA Synthesis Kit (Yeasen biotech Co., Ltd., China) according to the manufacturer's instructions. Quantitative Real-time PCR (qRT-PCR) reactions were performed with the Hieff Unicon® qPCR TaqMan Probe Master Mix (Yeasen biotech Co., Ltd., China) on a CFX96™ real-time PCR detection system (Bio-Rad, USA) and primers listed in Supplementary TABLE. Gene expression was calculated using the comparative 2 -ΔΔCT method with the actin for normalization. All PCR runs were performed in triplicate and the data analyzed by CFX Manager software (Bio-Rad, USA).

**mRNA stability assay**

The vector control CRC cells and 6PGD (or YTHDF2) knockdown cells or ALKBH5 overexpress cells were inoculated into 12-well plates. After being seeded for 24 hours, the cells were cultured in fresh medium with a density of 70–80%, then Actinomycin D (Act D， 5 μg/mL) was treated to cells for the indicated times, and the mRNA levels at each time point were analyzed by qPCR.

**Protein stability assay**

The vector control CRC cells and 6PGD knockdown cells or ALKBH5 overexpress cells were inoculated into 12-well plates. After being seeded for 24 hours, the cells were cultured in fresh medium with a density of 70–80%, then Cycloheximide (CHX, 5 mg/mL) was treated to cells for the indicated times, and the protein levels at each time point were analyzed by western blotting.

**Transwell assay and wound healing assay**

To detect the migration and invasion abilities of cells, Transwell assays (Corning, USA) and wound healing assays were performed. Then, 300 μL medium without serum suspended with cells was seeded into the upper chamber, and 900 μL medium with 20% FBS was filled with the bottom chamber. Migrated cells were stained with crystal violet (Solarbio, China) and then photographed. For wound healing assays, cells were cultured for growth in 6- well plates in FBS-withdrawn RPMI-1640, and scratches were generated by pipette tip. Migration of cells was quantified by photographs taken at 0 and 24 h after wounding.

**Flag-pull down (GST-pull down) or immunoprecipitation assay**

Cells were lysed in NP40 lysis buffer on ice 30 minutes and then centrifuged at 12,000 rpm for 15 minutes at 4°C. Subsequently, the cell lysates were subjected to immunoprecipitation using different antibody-conjugated beads (Flag-conjugated beads or GST-conjugated beads), which were incubated overnight at 4°C. Immune complexes were extensively washed for three times with cold TBS and analyzed by western blotting with specific antibodies.

**Immunohistochemical (IHC) staining**

Immunohistochemistry was performed on paraffin-embedded sections. Tissue sections were dewaxed and rehydrated using standard protocol. Antigen retrieval was performed by boiling samples in citrate buffer for 45 minutes. Endogenous peroxidase activity was inhibited by using 3% hydrogen peroxidase. Sections were blocked in 3% BSA in PBS and incubated in primary antibody (Ki67, 6PGD, MDM2, CCNA2 or HMGA2) overnight at 4°C. Sections were rinsed in PBS and developed using DAB. Sections were counterstained with hematoxylin. Quantification of staining was performed using Image J software.

**Immunofluorescence assay**

For immunofluorescence, cells were cultured on coverslips for 24 hours before experimental treatment. Samples were first fixed with 4% paraformaldehyde (15 minutes), rinsed three times with PBS, permeabilized with 0.1%Triton X-100 in PBS (20 minutes) and then blocked with 2% BSA in PBS (60 minutes). The samples were incubated with indicated primary antibodies made up in 2% BSA in PBS overnight at 4°C and rinsed three times with PBS prior to 30 minutes incubation with secondary antibodies conjugated to Alexa Fluor-555. After being washed three times with PBS, the coverslips were mounted using DAPI Fluoromount-G. Fluorescent micrographs were obtained using laser scanning confocal microscopy (Leica, Germany).

**Microscale thermophoresis (MST) assay**

The ALKBH5 protein was purchased from Proteintech, which was fluorescently labelled with NT-647 Kit for 30 minutes in the dark. After the fluorescent dye was eluted, the tagged ALKBH5 was collected. Then, a 16 step 2-fold dilution of 6PGD protein was performed and mixed with labeled ALKBH5. The samples were loaded into hydrophobic capillaries and the data was analyzed using MO. Affinity Analysis software. The dissociation constant KD was obtained by plotting the normalized fluorescence Fnorm against the logarithm of the different concentrations of the dilution series resulting in a sigmoid binding curve.

To investigate the competitive binding of 6PGD and m6A-modified RNA to ALKBH5, and confirm whether the binding region of 6PGD to ALKBH5 is the region where mRNA binds to ALKBH5, we synthesized a piece of RNA with two m6A modification motif(5’-3’: CGUGGACUCGGACUGGCU). A 16 step 2-fold dilution of 6PGD protein was performed, which was mixed with labeled ALKBH5 and 12μM RNA. The samples were loaded into hydrophobic capillaries and the data was analyzed using MO. Affinity Analysis software, by which we found that no binding of 6PGD and ALKBH5 was observed after added 12μM RNA to ALKBH5.

**SUPPLEMENTAL FIGURES AND LEGENDS**


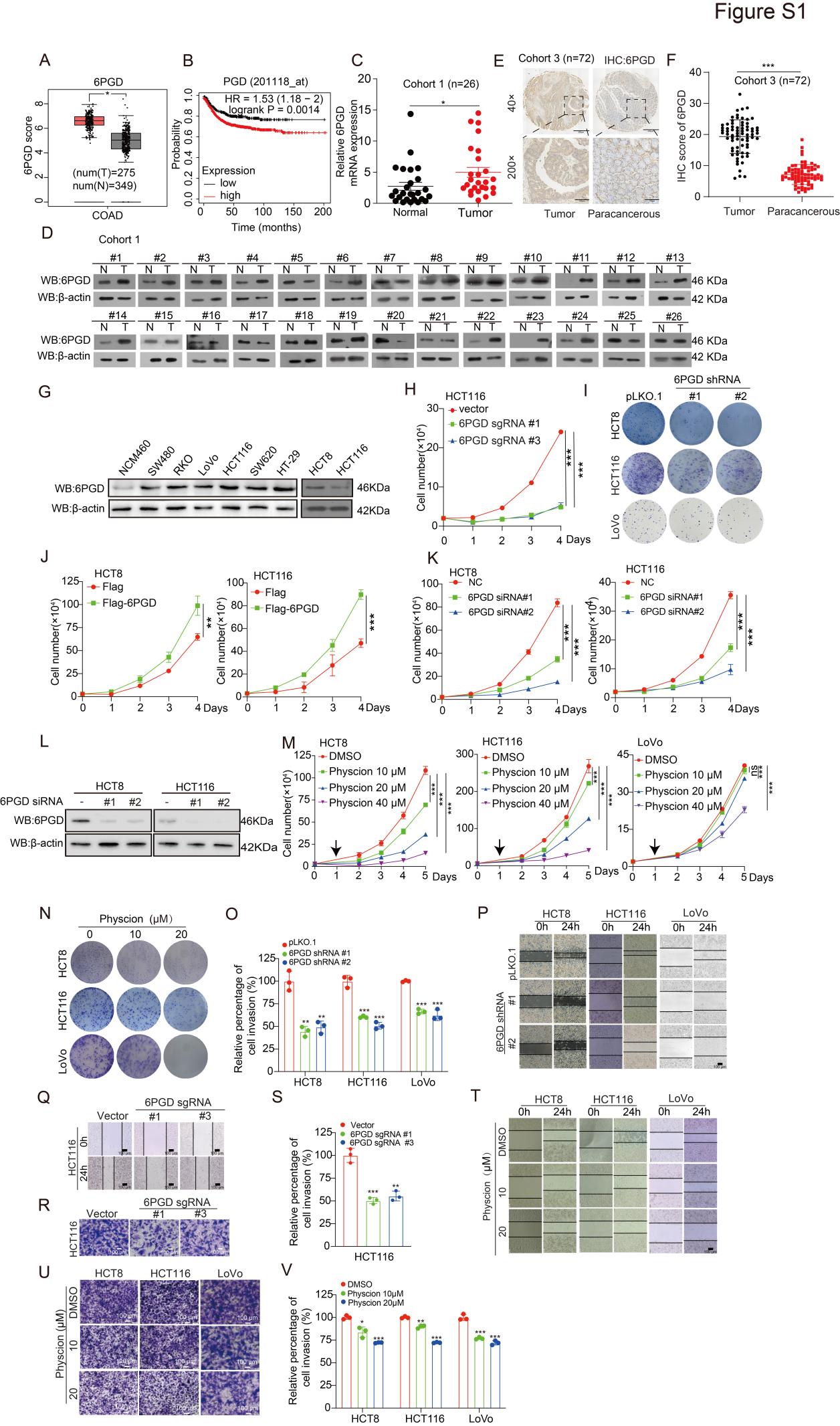


**Figure S1. 6PGD expression is elevated in colorectal cancer and required for colorectal cancer cell growth and metastasis.** (A) The expression of 6PGD mRNA was analyzed between CRC cancer tissues and normal tissues based on the GEPIA database. (B) Kaplan-Meier curves of overall survival in CRC cancer patients with high and low expression of 6PGD, calculated from (http://kmplot.com/analysis/). (C) The expression of 6PGD mRNA level in CRC tumor tissues (T) and matched adjacent normal tissues (N) (Cohort 1: n=26) were examined by qRT-PCR. (D) The protein level of 6PGD in CRC tumor tissues (T) and matched adjacent normal tissues(N) (Cohort 1: n=26) were examined by western blotting. (E) The expression of 6PGD was determined in CRC tissues microarray (TMA), which containing CRC tumor tissues (T) and adjacent normal tissues (Paracancerous) (Cohort 3: n=72) by immunohistochemical (IHC) stating assay. (F) Quantification of 6PGD expression by Image J software from IHC data. (G) The protein level of 6PGD in a panel of diverse human colorectal cancer cells, including SW480, RKO, LoVo, HCT116, SW620, HT-29, HCT8, and normal proliferating Human normal colonic epithelial cells [NCM460](https://www.kerafast.com/product/3039/human-bronchial-epithelial-cell-line-beas-2b) were examined by western blotting. (H) Cell proliferation was determined by cell number counting assay in HCT116 cells with knockout of 6PGD . (I) Cell proliferation was determined by colony formation in CRC cells with knockdown of 6PGD. (J) Cell proliferation was determined by cell number counting assay in HCT8 and HCT116 cells with exogenous expression of 6PGD. (K) Cell proliferation was determined by cell number counting assay in HCT8 and HCT116 cells with transient knockdown of 6PGD by siRNA. (L) The protein level of 6PGD were examined by western blotting in the knockdown of 6PGD HCT8 and HCT116 cells by siRNA. (M) Cell proliferation was determined by cell number counting assay in HCT8, HCT116 and LoVo cells treated with Physcion. (N) Cell proliferation was determined by colony formation in HCT8, HCT116, and LoVo cells treated with Physcion. (O) Quantification of cell migration by Image J software from Transwell invasion assay. (P) Cell migration was determined in HCT8, HCT116, and LoVo cells with stable knockdown of 6PGD by Wound-healing assay. (Q-S) Cell migration and invasion were determined in HCT116 cells with stable knockout of 6PGD by Wound-healing assay and Transwell invasion assay. (T-V) Cell migration and invasion were determined in HCT8, HCT116 and LoVo cells treated with Physcion by Wound-healing assay and Transwell invasion assay. The data represent mean values ± SD from three replicates of each sample (*0.01 < p < 0.05; **0.001 < p < 0.01; ***p < 0.001).


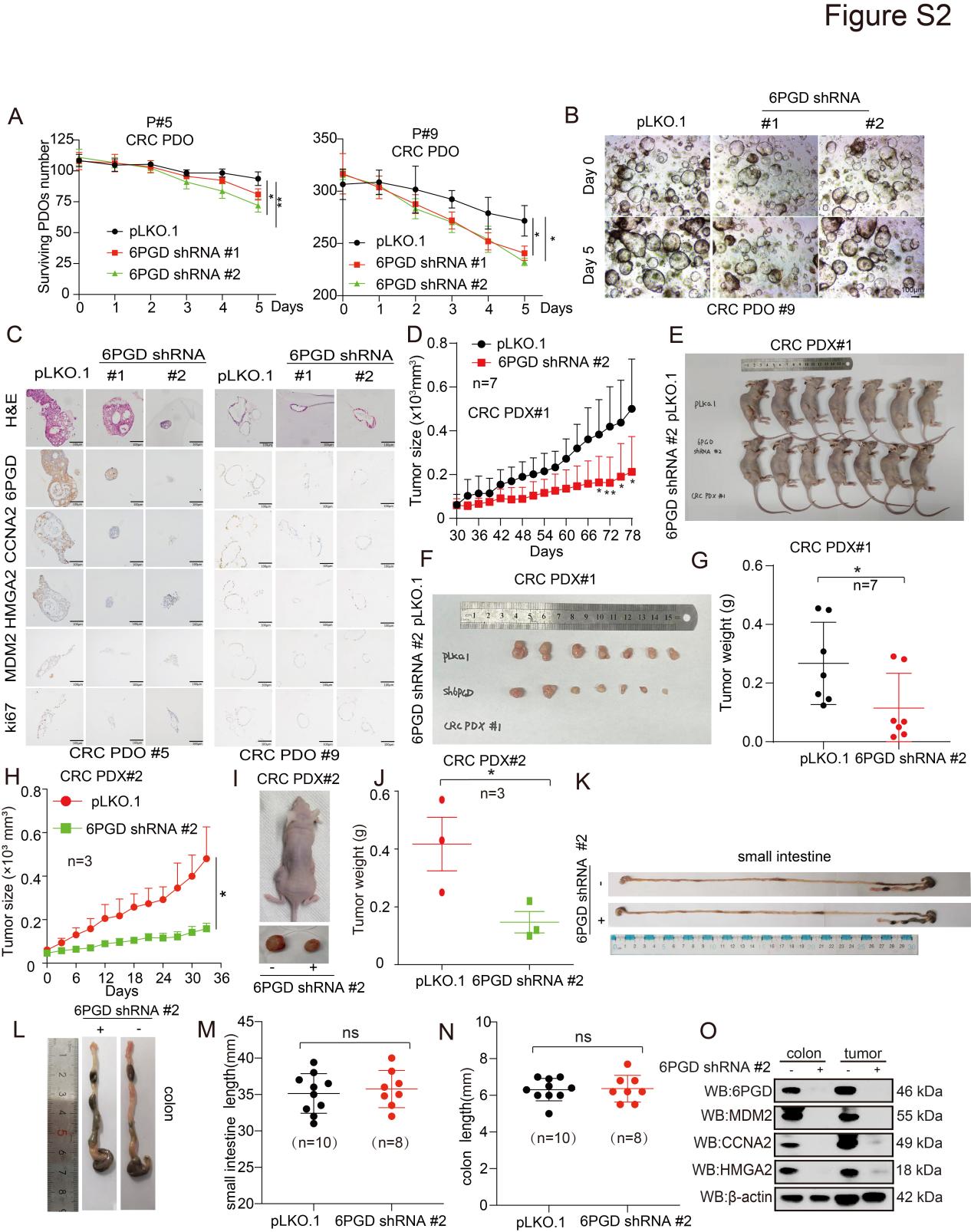


**Figure S2. 6PGD regulates colorectal cancer cell growth and metastasis.** (A) PDO tumor growth was determined in CRC PDO tumor with knockdown of 6PGD by PDO number counting assay. (B) Representative images of organoids with knockdown of 6PGD. (C) H&E, 6PGD, MDM2, CCNA2, HMGA2, and Ki67 were analyzed in knockdown of 6PGD PDO tumor tissues by IHC stating. (D) Tumor growth was compared between xenograft nude mice bearing with CRC PDX#1 tumor injected with 6PGD shRNA virus and control shRNA virus (n=7). (E) The nude mice are shown (PDX#1). (F) The tumors are shown (PDX#1). (J) Tumor mass in xenograft nude mice injected with 6PGD shRNA virus and control shRNA virus (PDX#1). (H) Tumor growth was compared between xenograft nude mice bearing with CRC PDX#2 tumor injected with 6PGD shRNA virus and control shRNA virus (n=3). (I) Dissected tumors in a representative nude mice are shown (PDX#2). (J) Tumor mass in xenograft nude mice injected with 6PGD shRNA virus and control shRNA virus (PDX#2). (K, L) Small intestine and colon in a pair of representative mouse models of induced colorectal cancer. (M, N) Small intestine length and colon length in induced colorectal cancer mice model which have injected with 6PGD shRNA virus and control shRNA virus. (O) The expression of 6PGD, MDM2, CCNA2, and HMGA2 protein were analyzed in induced CRC models tissues (T) and matched adjacent normal tissues (N) by western blotting. Data represent mean values ± SD from three replicates of each sample (*0.01 < p < 0.05; **0.001 < p < 0.01; ***p < 0.001).


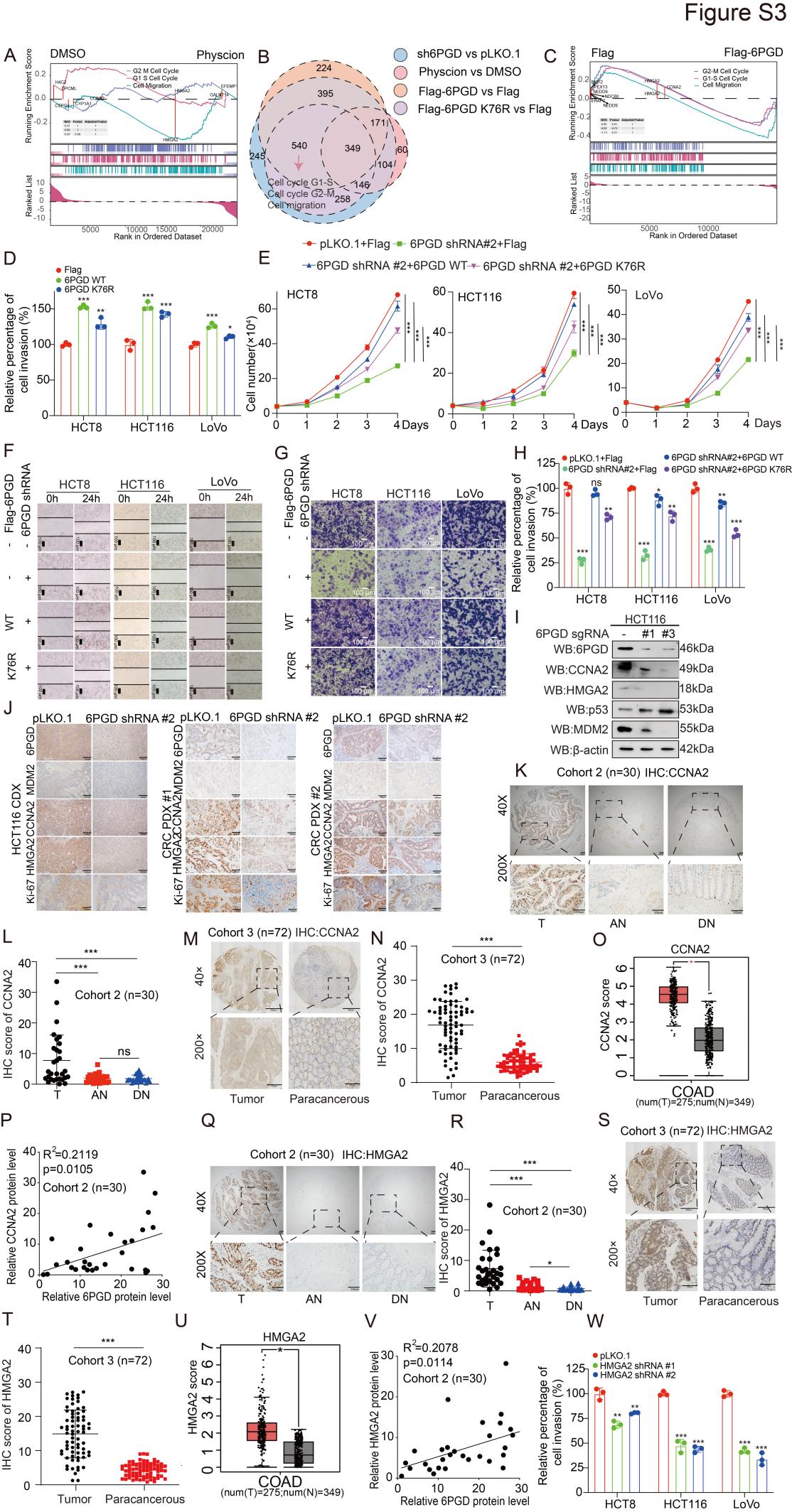
**Figure S3. 6PGD Promotes Colorectal cancer Progression in a Catalytic-Activity-Independent Manner.** (A) The cell cycle and cell migration pathways were enriched in 6PGD related pathway were analyzed by gene set enrichment analysis (GSEA) based on Physcion treated and control HCT116 cells RNA-seq. (B) Venn diagram showing a total of 540 shared regulation genes were observed among 6PGD knockdown, exogenous expression of 6PGD WT, and in Physcion treatment-related down-regulation genes. (C) The cell cycle and cell migration pathways were enriched in 6PGD related pathway were analyzed by GSEA based on exogenous expression of 6PGD WT and control HCT116 cells RNA-seq. (D) Cell invasion was determined in knockdown of 6PGD CRC cells with exogenous expression of 6PGD WT or 6PGD K76R by Transwell invasion assay. (E) Cell proliferation was determined in knockdown of 6PGD CRC cells with exogenous expression of 6PGD WT or 6PGD K76R by cell number counting assay. (F) Cell migration was determined in knockdown of 6PGD CRC cells with exogenous expression of 6PGD WT or 6PGD K76R by Wound-healing assay. (G, H) Cell invasion was determined in knockdown of 6PGD CRC cells with exogenous expression of 6PGD WT or 6PGD K76R by Transwell invasion assay. (I) The expression of 6PGD, CCNA2, HMGA2, p53, and MDM2 were determined in knockout of 6PGD cells by western blotting. (J) 6PGD, MDM2, CCNA2, HMGA2, and Ki67 were analyzed in knockdown of 6PGD CDX/PDX#1/PDX#2 tumor by IHC. (K) The expression of CCNA2 was determined in CRC tissues microarray (TMA), which containing distant normal tissues (DN), adjacent normal tissues (AN) and CRC tumor tissues (T) (Cohort 2: n=30) by immunohistochemical (IHC) assay. (L) Quantification of CCNA2 expression by Image J software from IHC data (Cohort 2: n=30). (M) The expression of CCNA2 was determined in CRC tissues microarray (TMA), which containing adjacent normal tissues (Paracancerous) and CRC tumor tissues (T) (Cohort 3: n=72) by IHC assay. (N) Quantification of CCNA2 expression by Image J software from IHC data (Cohort 3: n=72).(O) The levels of CCNA2 mRNA were analyzed between CRC cancer tissues and normal tissues based on GEPIA database. (P) Correlation of 6PGD and CCNA2 protein levels were analyzed in 30 clinical colon cancer tissues based on IHC staining score. (Q) The expression of HMGA2 was determined by immunohistochemical (IHC) assay in CRC tissues microarray (TMA), which containing distant normal tissues (DN), adjacent normal tissues (AN) and CRC tumor tissues (T) (Cohort 2: n=30). (R) Quantification of HMGA2 expression by Image J software from IHC data (Cohort 2: n=30). (S) The expression of HMGA2 was determined by IHC assay in CRC tissues microarray (TMA), which containing adjacent normal tissues (Paracancerous) and CRC tumor tissues (T) (Cohort 3: n=72). (T) Quantification of HMGA2 expression by Image J software from IHC data (Cohort 3: n=72). (U) The levels of HMGA2 mRNA were analyzed between CRC cancer tissues and normal tissues from GEPIA database. (V) Correlation of 6PGD and HMGA2 protein levels were analyzed in 30 clinical colon cancer tissues based on IHC staining score. (W) Cell invasion was determined in the knockdown of HMGA2 CRC cells by Transwell invasion assay. Data represent mean values ± SD from three replicates of each sample (*0.01 < p < 0.05; **0.001 < p < 0.01; ***p < 0.001).


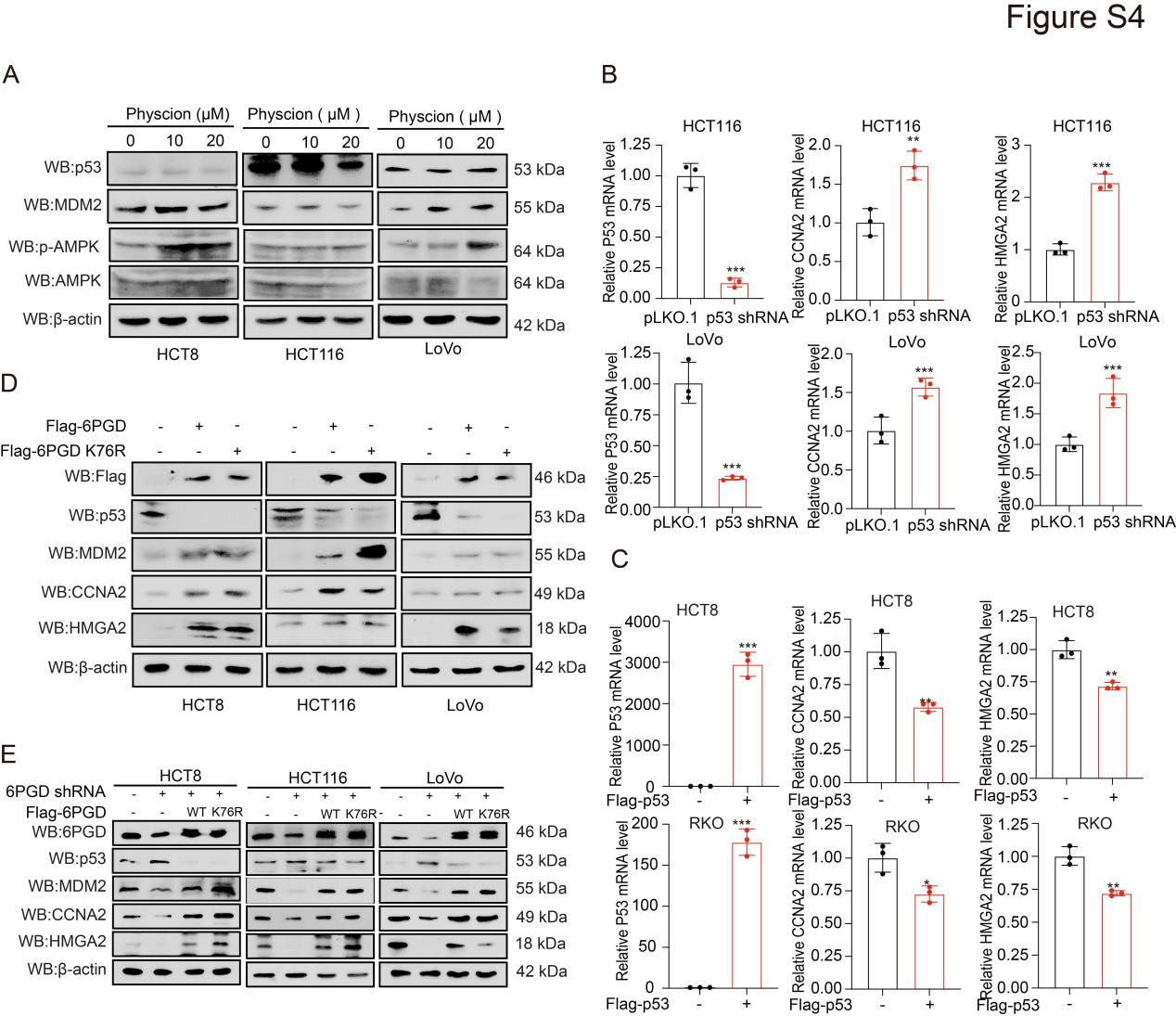


**Figure S4. 6PGD non-enzymatic activity dependent on p53 to Modulates the expression of CCNA2 and HMGA2.** (A) The expression of 6PGD, p53 and MDM2 were determined in HCT8, HCT116 and LoVo cells treated with Physcion by western blotting. (B) The expression of CCNA2 and HMGA2 were determined in the knockdown of p53 or vector control CRC cells by qRT-PCR. (C) The expression of CCNA2 and HMGA2 were determined in CRC cells with exogenous expression of p53 by qRT-PCR. (D) The p53, MDM2, CCNA2, and HMGA2 protein levels were determined in CRC cells with exogenous expression of 6PGD WT or 6PGD K76R by western blotting. (E) The expression of p53, MDM2, CCNA2, and HMGA2 were determined in the knockdown of 6PGD cells with exogenous expression of 6PGD WT or 6PGD K76R by western blotting. The data represent mean values ± SD from three replicates of each sample (*0.01 < p < 0.05; **0.001 < p < 0.01; ***p < 0.001).


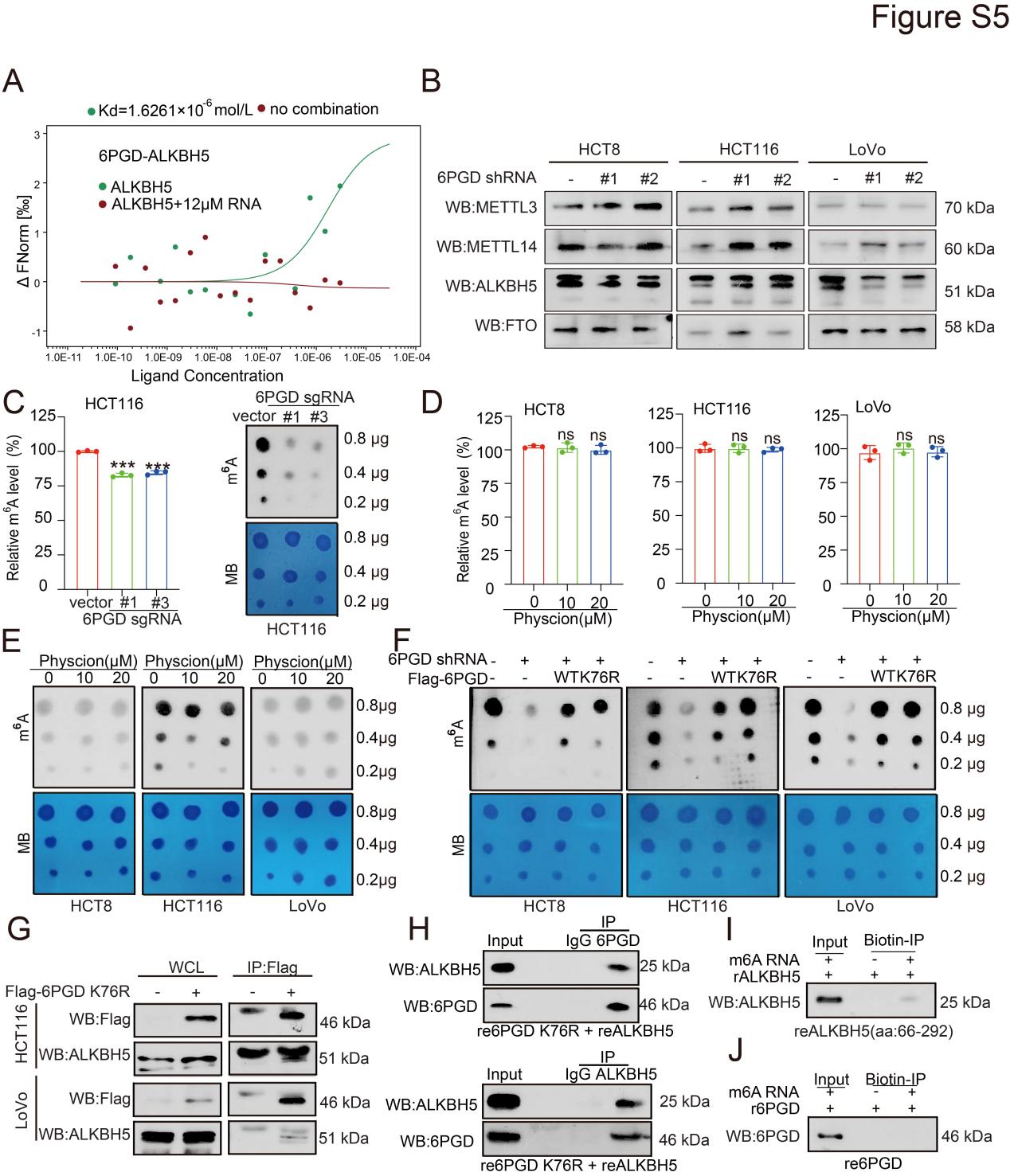


**Figure S5. 6PGD regulates the m^6^A level in a Catalytic-Activity-Independent Manner.** (A) The affinity of ALKBH5 and 6PGD was determined by microscale thermophoresis (MST) assays. (B) The expression of m^6^A-related enzymes were determined in knockdown of 6PGD CRC cells by western blotting. The actin was same as Figure 2J. (C) The m^6^A levels were determined by the m6A RNA Methylation Quantification Kit (Colorimetric) in the knockout of 6PGD or vector control CRC cells (*left)*. The m^6^A levels were determined by dot blotting assay in the knockout of 6PGD or vector control CRC cells (*right)*. (D) The m^6^A levels were determined by the m^6^A RNA Methylation Quantification Kit (Colorimetric) in CRC cells treated with the indicated dose of Physcion. (E) The m^6^A levels were determined by dot blotting assay in CRC cells treated with the indicated dose of Physcion. (F) The m^6^A levels were determined by dot blotting assay in knockdown of 6PGD CRC cells with exogenous expression of 6PGD WT or 6PGD K76R. (G) The interaction between Flag-6PGD K76R and endogenous ALKBH5 were determined in HCT116 and LoVo cells by *Flag-pull down* assay. (H) The interaction between recombinant 6PGD K76R and recombinant ALKBH5 were determined by *in vitro* Co-IP assay. (I) The *in vitro* interaction between recombinant ALKBH5 and biotin-RNA were examined by biotin-pull down. ALKBH5 associates with RNA in vitro, biotin-RNA was incubated with reALKBH5, after biotin-Immunoprecipitation (biotin-IP), the interaction of RNA with reALKBH5 were analyzed by western blotting. (J) The *in vitro* interaction between recombinant 6PGD and biotin-RNA were examined by biotin-pull down. 6PGD associates with RNA *in vitro*, biotin-RNA was incubated with re6PGD, after biotin-Immunoprecipitation (biotin-IP), the interaction of RNA with re6PGD were analyzed by western blotting. The data represent mean values ± SD from three replicates of each sample (*0.01 < p < 0.05; **0.001 < p < 0.01; ***p < 0.001).


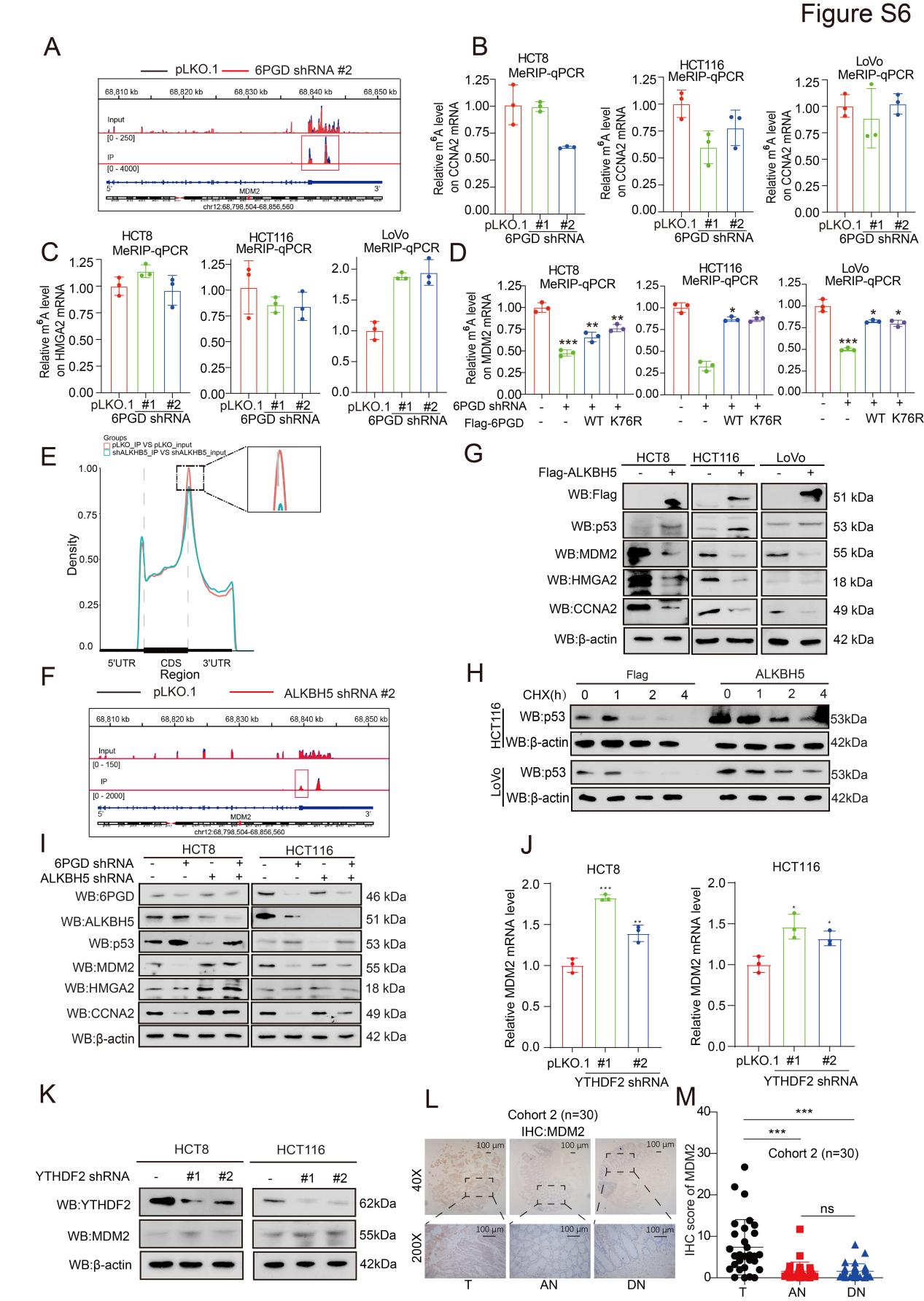


**Figure S6. 6PGD regulates the m^6^A level on MDM2 mRNA mediated by ALKBH5.** (A) The relative abundance of m^6^A sites along MDM2 mRNA in the knockdown of 6PGD cells and control cells were analyzed by m^6^A-seq. (B, C) The m^6^A level on CCNA2 mRNA (B) and HMGA2 mRNA (C) in knockdown of 6PGD cells were examined by the MeRIP-qPCR. (D) The MeRIP-qPCR analysis of MDM2 m^6^A levels in 6PGD knockdown cells with exogenous expression of 6PGD WT or 6PGD K76R. (E) The density distribution of m^6^A peaks in messenger RNA (mRNA) transcripts based on knockdown of 6PGD m6A-seq. (F) The relative abundance of m^6^A sites along MDM2 mRNA in the knockdown of ALKBH5 cells and control cells were analyzed by m^6^A-seq. (G) The p53, MDM2, HMGA2, and CCNA2 protein levels were determined in exogenous expression ALKBH5 cells by western blotting. (H) The protein stability of p53 was determined by western blot in HCT116 and LoVo cells with exogenous expression ALKBH5. (I) The expression of p53, MDM2, CCNA2, and HMGA2 were determined in knockdown of 6PGD cells with knockdown of ALKBH5 by western blotting. (J) The m^6^A level on MDM2 mRNA in knockdown of YTHDF2 cells were examined by the MeRIP-qPCR. (K) The YTHDF2 and MDM2 protein levels were determined in knockdown of YTHDF2 cells by western blotting. (L) The expression of MDM2 was determined in CRC tissues microarray (TMA), which containing distant normal tissues (DN), adjacent normal tissues (AN) and CRC tumor tissues (T) (Cohort 2: n=30) by immunohistochemical (IHC) assay. (M) Quantification of MDM2 expression by Image J software from based on IHC stating. The data represent mean values ± SD from three replicates of each sample (*0.01 < p < 0.05; **0.001 < p < 0.01; ***p < 0.001).


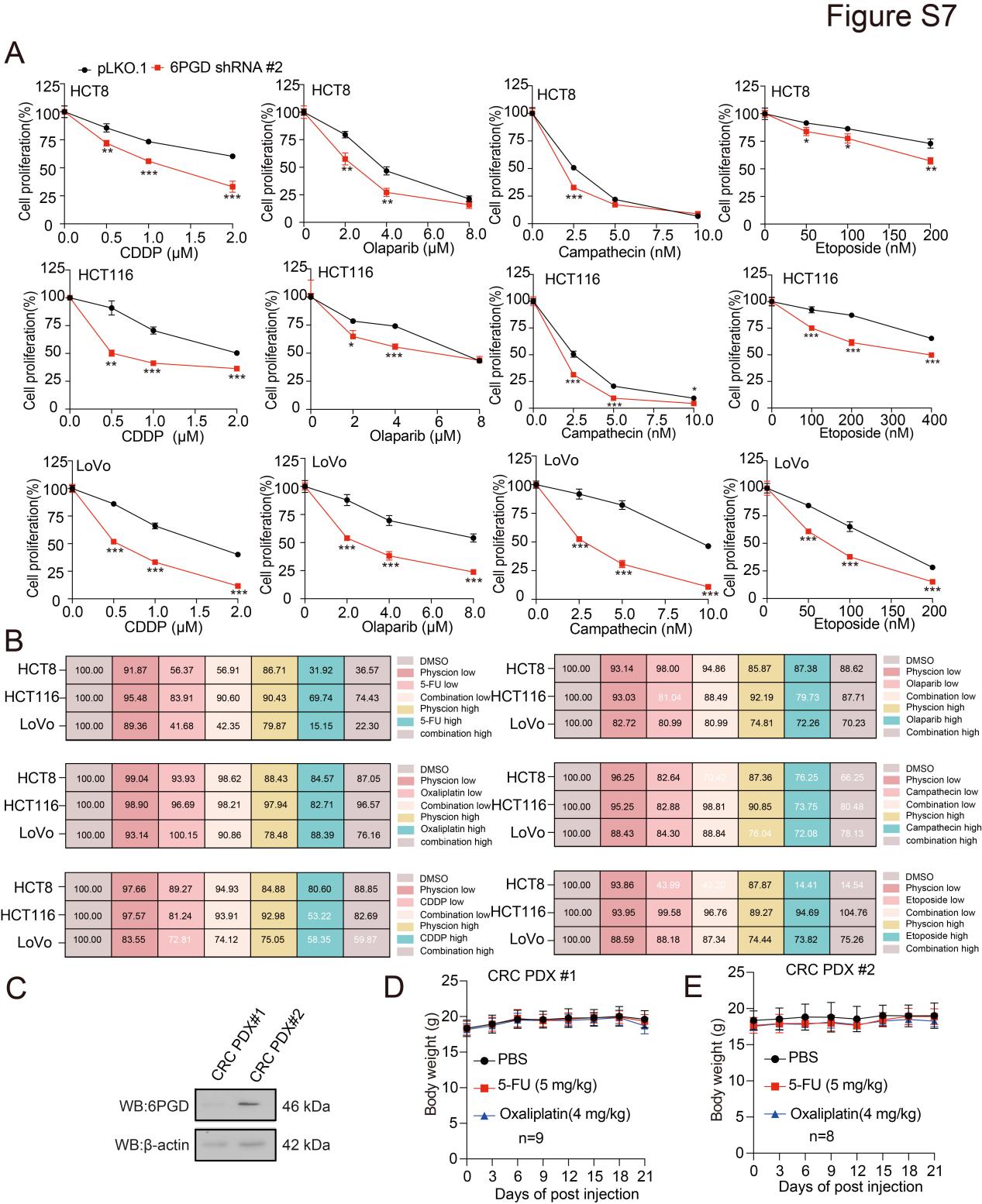


**Figure S7. Supression of 6PGD expression can enhance chemotherapy sensitivity.** (A) Cell proliferation was determined by cell number counting assay in CRC cells with stable knockdown of 6PGD when treated with or without the indicated dose of chemotherapeutic drugs. (B) Cell proliferation was determined by cell number counting in CRC cells treated with Physcion, or chemotherapeutic, alone and combination. (C) The expression of 6PGD protein was determined in PDX#1 and PDX#2 tumor tissues by western blotting. (D) The body weights of mice bearing with CRC PDX#1 injection with 5-FU or Oxaliplatin (n = 9). (E) The body weights of mice bearing with CRC PDX#2 injection with 5-FU or Oxaliplatin (n = 8). The data represent mean values ± SD from three replicates of each sample (*0.01 < p < 0.05; **0.001 < p < 0.01; ***p < 0.001).

**Supplementary Table 1.** **Expression of 6PGD protein in CRC**

| **Diagnosis** | **No.of case** | **6PGD** | | | | **Positive cases rate (%)** | **strong positive cases rate(%)** |
| --- | --- | --- | --- | --- | --- | --- | --- |
|  |  | **-** | **+** | **++** | **+++** |  |  |
| CRC | 30 | 4 | 12 | 14 | 0 | 86.7%*** | **46.7%***** |
| Adjacent normal colon  Distant normal colon | 30  30 | 6  18 | 22  10 | 2  2 | 0  0 | 80.0%  40.0% | 6.7%  6.7% |

**Positive rate**: percentage of positive cases with +, ++, and +++ staining score.

**Strongly positive rate** (high-level expression): percentage of positive cases with ++ and +++ staining score.

*** *p*<0.0001 compared with normal colon.

**Supplementary Table 2. Relationship between 6PGD protein overexpression and the clinicopathological features of CRC**

| **Variables** | **No. of case (*n*)** | **6PGD strong positive rate (%)** | ***P* value** |
| --- | --- | --- | --- |
| **Gender**  Male  Female  **Age (years)**  >62  ≤62  **Tumor size**  ≤4 cm  ＞4cm  **Differentiation grade**  Low and low-middle  Middle and high  **TNM stage**  Ⅰa  Ⅲa  Ⅲb  **Duke’s stage**  B  C | 20  10  13  17  15  15  9  21  0  23  7  0  30 | 10(50.0%)  4(40.0%)  8(61.5%)  6(35.3%)  5(33.3%)  9(60.0%)  5(55.6%)  9 (42.9%)  0(0.0%)  11(47.8%)  3(42.9%)  0(0.0%)  14(46.7%) | 0.7475  0.9478  0.9386  0.9480  N/A  N/A |

**­**

**Supplementary Table 3. The cell cycle was detected by knocking down 6PGD**

| HCT8 | G0/G1 | S | G2/M | PI |
| --- | --- | --- | --- | --- |
| pLKO.1 | 48.36±0.57 | 37.91±0.06 | 13.72±0.59 | 51.63±0.57 |
| 6PGD shRNA #1 | 55.17±1.55 | 32.24±1.34 | 12.59±0.86 | 44.83±1.56 |
| 6PGD shRNA #2 | 53.59±1.18 | 33.11±1.27 | 13.29±0.18 | 46.41±1.18 |
|  |  |  |  |  |
| HCT116 | G0/G1 | S | G2/M | PI |
| pLKO.1 | 40.75±0.85 | 46.44±0.67 | 12.81±0.64 | 59.25±0.85 |
| 6PGD shRNA #1 | 48.84±0.54 | 33.71±1.36 | 17.44±0.89 | 51.15±0.54 |
| 6PGD shRNA #2 | 46.88±1.04 | 32.94±2.77 | 20.17±1.01 | 53.12±1.04 |
|  |  |  |  |  |
| LoVo | G0/G1 | S | G2/M | PI |
| pLKO.1 | 41.82±2.06 | 35.48±0.74 | 23.7±0.4 | 58.61±1.46 |
| 6PGD shRNA #1 | 61.6±1.01 | 14.47±0.42 | 23.93±0.72 | 39.01±1.23 |
| 6PGD shRNA #2 | 59.16±1.05 | 14.77±1.2 | 26.06±0.26 | 40.83±1.05 |
|  |  |  |  |  |
| HCT116 | G0/G1 | S | G2/M | PI |
| vector | 37.00±0.88 | 39.61±0.33 | 23.39±0.79 | 63.00±0.88 |
| 6PGD sgRNA #1 | 49.59±0.54 | 27.13±0.38 | 23.28±0.44 | 50.41±0.54 |
| 6PGDsgRNA #3 | 50.24±0.54 | 25.93±1.24 | 23.82±0.73 | 49.76±0.54 |
| PI=[(S+G2/M)/(G0/G1+S+G2/M)*100 | | | | |
| PI=Proliferation Index | | | | |

**Supplementary Table 4. The cell cycle was detected by overexpress 6PGD-WT or 6PGD-K76R**

| HCT8 | G0/G1 | S | G2/M | PI |
| --- | --- | --- | --- | --- |
| Flag | 47.02±0.33 | 18.79±0.71 | 34.19±0.53 | 52.98±0.33 |
| 6PGD WT | 41.54±0.16 | 26.88±0.63 | 31.59±0.58 | 58.46±0.16 |
| 6PGD K76R | 43.30±0.30 | 25.20±0.23 | 31.49±0.18 | 56.70±0.30 |
|  |  |  |  |  |
| HCT116 | G0/G1 | S | G2/M | PI |
| Flag | 41.11±0.41 | 30.46±0.41 | 28.43±0.39 | 58.89±0.41 |
| 6PGD WT | 35.55±0.97 | 41.16±2.42 | 22.83±1.46 | 64.44±0.98 |
| 6PGD K76R | 37.57±0.18 | 37.52±0.42 | 24.92±0.58 | 62.43±0.58 |
|  |  |  |  |  |
| LoVo | G0/G1 | S | G2/M | PI |
| Flag | 67.20±0.75 | 13.39±0.61 | 19.40±0.46 | 32.8±0.46 |
| 6PGD WT | 48.07±1.01 | 36.88±0.63 | 15.05±1.56 | 51.93±1.01 |
| 6PGD K76R | 53.24±0.71 | 30.82±0.36 | 15.94±0.50 | 46.76±0.71 |
| PI=[(S+G2/M)/(G0/G1+S+G2/M)*100 | | | | |
| PI=Proliferation Index | | | | |

**Supplementary Table 5. The cell cycle was detected by overexpress 6PGD-WT or 6PGD-K76R in 6PGD knockdown cells**

| HCT8 | G0/G1 | S | G2/M | PI |
| --- | --- | --- | --- | --- |
| pLKO.1+Flag | 42.12±0.10 | 43.67±0.04 | 23.20±0.09 | 57.88±0.10 |
| 6PGD shRNA+Flag | 56.52±0.52 | 27.30±0.65 | 16.17±1.06 | 43.48±0.52 |
| 6PGD shRNA+6PGD WT | 48.62±1.07 | 36.60±0.67 | 14.68±0.46 | 51.38±1.07 |
| 6PGD shRNA+6PGD K76R | 55.18±0.16 | 26.18±0.94 | 18.63±1.03 | 44.81±0.16 |
|  |  |  |  |  |
| HCT116 | G0/G1 | S | G2/M | PI |
| pLKO.1+Flag | 43.43±0.87 | 42.98±4.16 | 13.70±3.37 | 56.68±0.87 |
| 6PGD shRNA+Flag | 53.64±2.42 | 32.30±1.06 | 14.05±1.61 | 46.36±2.42 |
| 6PGD shRNA+6PGD WT | 41.30±0.82 | 40.88±1.08 | 17.82±0.59 | 58.70±0.82 |
| 6PGD shRNA+6PGD K76R | 48.00±0.52 | 36.20±1.96 | 15.81±2.08 | 52.00±0.52 |
|  |  |  |  |  |
| LoVo | G0/G1 | S | G2/M | PI |
| pLKO.1+Flag | 45.01±0.50 | 37.74±0.18 | 17.25±0.32 | 54.99±0.50 |
| 6PGD shRNA+Flag | 59.93±0.61 | 23.56±0.33 | 16.51±0.29 | 40.07±0.61 |
| 6PGD shRNA+6PGD WT | 50.17±1.45 | 31.34±0.29 | 18.82±0.61 | 50.00±1.16 |
| 6PGD shRNA+6PGD K76R | 55.99±0.62 | 26.30±0.43 | 17.71±0.19 | 44.00±0.62 |
| PI=[(S+G2/M)/(G0/G1+S+G2/M)*100 | | | | |
| PI=Proliferation Index | | | | |

**Supplementary Table 6.** **Expression of CCNA2 protein in CRC**

| **Diagnosis** | **No.of case** | **CCNA2** | | | | **Positive cases rate (%)** | **strong positive cases rate(%)** |
| --- | --- | --- | --- | --- | --- | --- | --- |
|  |  | **-** | **+** | **++** | **+++** |  |  |
| CRC | 30 | 21 | 6 | 3 | 0 | 30.0%*** | **10.0%***** |
| Adjacent normal colon  Distant normal colon | 30  30 | 30  30 | 0  0 | 0  0 | 0  0 | 0  0 | 0  0 |

**Positive rate**: percentage of positive cases with +, ++, and +++ staining score.

**Strongly positive rate** (high-level expression): percentage of positive cases with ++ and +++ staining score.

**** *p*<0.0001 compared with adjacent normal colon.

**Supplementary Table 7. Relationship between CCNA2 protein overexpression and the clinicopathological features of CRC**

| **Variables** | **No. of case (*n*)** | **CCNA2 strong positive rate (%)** | ***P* value** |
| --- | --- | --- | --- |
| **Gender**  Male  Female  **Age (years)**  >62  ≤62  **Tumor size**  ≤4 cm  ＞4cm  **Differentiation grade**  Low and low-middle  Middle and high  **TNM stage**  Ⅰa  Ⅲa  Ⅲb  **Duke’s stage**  B  C | 20  10  13  17  15  15  9  21  0  23  7  0  30 | 1(5.0%)  2(20.0%)  2(15.4%)  1(5.9%)  1(6.7%)  2(13.3%)  1(11.1%)  2 (9.5%)  0(0.0%)  2(8.7%)  1(14.3%)  0(0.0%)  3(10.0%) | 0.1695  0.4180  0.2356  0.0324  N/A  N/A |

**­**

**Supplementary Table 8. The cell cycle was detected by knocking down CCNA2**

| HCT8 | G0/G1 | S | G2/M | PI |
| --- | --- | --- | --- | --- |
| pLKO.1 | 40.86±0.89 | 27.43±0.21 | 31.71±0.78 | 59.14±0.89 |
| CCNA2 shRNA | 50.31±0.85 | 21.83±3.21 | 27.82±2.31 | 49.67±0.88 |
|  |  |  |  |  |
| HCT116 | G0/G1 | S | G2/M | PI |
| pLKO.1 | 30.16±1.53 | 23.31±1.46 | 46.52±1.42 | 69.83±1.53 |
| CCNA2 shRNA | 23.53±1.39 | 35.61±1.25 | 40.83±1.61 | 76.47±1.38 |
|  |  |  |  |  |
| LoVo | G0/G1 | S | G2/M | PI |
| pLKO.1 | 42.74±0.45 | 28.31±0.34 | 28.95±0.76 | 57.26±0.45 |
| CCNA2 shRNA | 25.87±1.15 | 59.54±2.1 | 14.59±1.01 | 74.13±1.15 |
| PI=[(S+G2/M)/(G0/G1+S+G2/M)*100 | | | | |
| PI=Proliferation Index | | | | |

**Supplementary Table 9.** **Expression of HMGA2 protein in CRC**

| **Diagnosis** | **No.of case** | **HMGA2** | | | | **Positive cases rate (%)** | **strong positive cases rate(%)** |
| --- | --- | --- | --- | --- | --- | --- | --- |
|  |  | **-** | **+** | **++** | **+++** |  |  |
| CRC | 30 | 22 | 6 | 2 | 0 | 26.7%*** | **6.7%***** |
| Adjacent normal colon  Distant normal colon | 30  30 | 30  30 | 0  0 | 0  0 | 0  0 | 0  0 | 0  0 |

**Positive rate**: percentage of positive cases with +, ++, and +++ staining score.

**Strongly positive rate** (high-level expression): percentage of positive cases with ++ and +++ staining score.

**** *p*<0.0001 compared with adjacent normal colon.

**Supplementary Table 10. Relationship between HMGA2 protein overexpression and the clinicopathological features of CRC**

**­**

| **Variables** | **No. of case (*n*)** | **HMGA2 strong positive rate (%)** | ***P* value** |
| --- | --- | --- | --- |
| **Gender**  Male  Female  **Age (years)**  >62  ≤62  **Tumor size**  ≤4 cm  ＞4cm  **Differentiation grade**  Low and low-middle  Middle and high  **TNM stage**  Ⅰa  Ⅲa  Ⅲb  **Duke’s stage**  B  C | 20  10  13  17  15  15  9  21  0  23  7  0  30 | 2(10.0%)  0(0.0%)  1(7.7%)  1(5.9%)  0(0.0%)  2(13.3%)  1(11.1%)  1 (4.8%)  0(0.0%)  1(4.3%)  1(14.3%)  0(0.0%)  2(6.7%) | 0.3998  0.4939  0.4224  0.7266  N/A  N/A |

**Supplementary Table 11.** **Expression of MDM2 protein in CRC**

| **Diagnosis** | **No.of case** | **MDM2** | | | | **Positive cases rate (%)** | **strong positive cases rate(%)** |
| --- | --- | --- | --- | --- | --- | --- | --- |
|  |  | **-** | **+** | **++** | **+++** |  |  |
| CRC | 30 | 21 | 6 | 3 | 0 | 30.0%*** | **10.0%***** |
| Adjacent normal colon  Distant normal colon | 30  30 | 29  30 | 1  0 | 0  0 | 0  0 | 3.3%  0 | 0  0 |

**Positive rate**: percentage of positive cases with +, ++, and +++ staining score.

**Strongly positive rate** (high-level expression): percentage of positive cases with ++ and +++ staining score.

**** *p*<0.0001 compared with adjacent normal colon.

**Supplementary Table 12. Relationship between MDM2 protein overexpression and the clinicopathological features of CRC­**

| **Variables** | **No. of case (*n*)** | **MDM2 strong positive rate (%)** | ***P* value** |
| --- | --- | --- | --- |
| **Gender**  Male  Female  **Age (years)**  >62  ≤62  **Tumor size**  ≤4 cm  ＞4cm  **Differentiation grade**  Low and low-middle  Middle and high  **TNM stage**  Ⅰa  Ⅲa  Ⅲb  **Duke’s stage**  B  C | 20  10  13  17  15  15  9  21  0  23  7  0  30 | 0(0.0%)  3(30.0%)  1(7.7%)  2(11.8%)  0(0.0%)  3(20.0%)  0(0.0%)  3 (14.3%)  0(0.0%)  3(13.0%)  0(0.0%)  0(0.0%)  3(10.0%) | 0.4222  0.5692  0.7697  0.0292  N/A  N/A |
